# Total synthesis of leopolic acid A, a natural 2,3-pyrrolidinedione with antimicrobial activity

**DOI:** 10.3762/bjoc.12.159

**Published:** 2016-07-29

**Authors:** Atul A Dhavan, Rahul D Kaduskar, Loana Musso, Leonardo Scaglioni, Piera Anna Martino, Sabrina Dallavalle

**Affiliations:** 1 Department of Food, Environmental and Nutritional Sciences, Division of Chemistry and Molecular Biology, Università degli Studi di Milano, via Celoria 2, I-20133 Milano, Italy; 2 Department of Veterinary Medicine - Microbiology and Immunology, Università degli Studi di Milano, via Celoria 10, I-20133 Milano, Italy

**Keywords:** antimicrobial, heterocyclic compounds, natural products, pyrrolidinedione, total synthesis

## Abstract

The first total synthesis of leopolic acid A, a fungal metabolite with a rare 2,3-pyrrolidinedione nucleus linked to an ureido dipeptide, was designed and carried out. Crucial steps for the strategy include a Dieckmann cyclization to obtain the 2,3-pyrrolidinedione ring and a Wittig olefination to install the polymethylene chain. An oxazolidinone-containing leopolic acid A analogue was also synthesized. The antibacterial activity showed by both compounds suggests that they could be considered as promising candidates for future developments.

## Introduction

Great concern has recently been expressed about the diminishing efficacy of current antibiotic therapies and the emergence of multidrug resistant strains. Novel potent compounds endowed with new mechanisms of action are urgently needed. In this regard, natural products continue to be a rich source of biologically validated structures, which can be modified and optimized in drug discovery [1].

**Figure 1:**
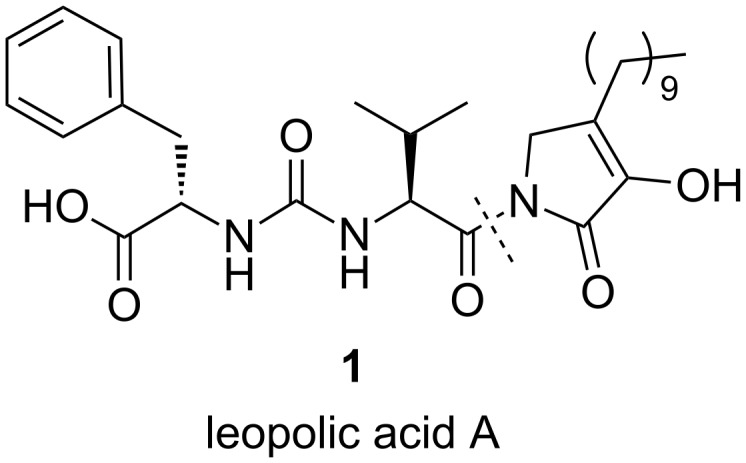
Structure of leopolic acid A.

Chemical analysis of a terrestrial-derived *Streptomyces* sp. isolated from the rhizosphere of the plant *Juniperus excelsa* yielded a new metabolite, leopolic acid A (**1**, Figure 1) [2]. Leopolic acid has unprecedented structural features consisting of an aliphatic side chain attached to a 2,3-pyrrolidinedione residue, which in turn, is connected to the ureido dipeptide L-Phe-L-Val. The compound showed antifungal and antibacterial activity against *Mucor hiemalis* and *Staphylococcus aureus* with a MIC of 32 and 16 μg/mL, respectively [2].

Compounds containing the isomeric 2,4-pyrrolidinedione ring system (tetramic acids) are widespread among the fungal metabolites and show a number of biological activities, i.e., antibacterial, antiviral, antifungal and anticancer. More than one hundred of them have been isolated from a variety of natural sources [3].

Conversely, natural compounds with a simple 2,3-pyrrrolidinedione nucleus are indeed very few [4–6]. Even though this core can be considered an attractive target, synthetic studies directed to find new biologically active compounds are scarce as well [7–9].

The unique structure and bioactivity of leopolic acid prompted us to plan a synthesis which might be used in the preparation of various analogues. A retrosynthetic disconnection of **1** at the amide bond gave two fragments, the ureido dipeptide L-Phe-L-Val and 4-decyl-3-hydroxy-1,5-dihydropyrrol-2-one, which appeared well suited for a convergent synthetic approach (Figure 1).

## Results and Discussion

**Scheme 1:**
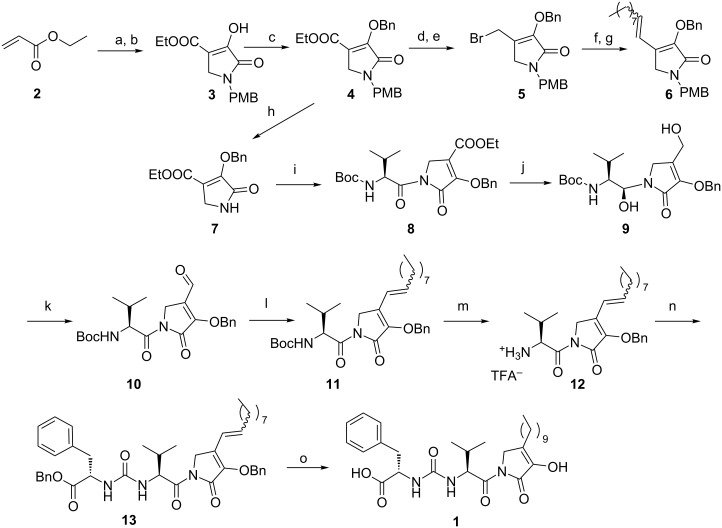
Synthesis of leopolic acid A. Reagents and conditions: a) *p*-methoxybenzylamine, EtOH, rt, 12 h, 98%; b) diethyl oxalate, NaOEt, EtOH, reflux, 3 h, 83%; c) BnBr, K_2_CO_3_, DMF, 0 °C to rt, 1 h, 50%; d) DIBAL-H, CH_2_Cl_2_, −78 °C, 2 h, 56%; e) PPh_3_, CBr_4_, CH_2_Cl_2_, rt, 5 h, 52%; f) PPh_3_, toluene, reflux, 5 h, 75%; g) *n*-nonanal, LiHMDS, THF, −78 °C to rt, 5 h, 52%; h) CAN, CH_3_CN:H_2_O, 0 °C to rt, 3 h, 73%; i) 2-*tert*-butoxycarbonylamino-3-methylbutyric acid pentafluorophenyl ester, *n*-BuLi, THF, −78 °C, 0.5 h, 71%; j) DIBAL-H, CH_2_Cl_2_, −78 °C, 2 h, 30%; k) PCC, CH_2_Cl_2_, 0 °C to rt, 12 h, 46%; l) *n*-nonyltriphenylphosphonium bromide, *n*-BuLi, THF, −78 °C to 0 °C, 2 h; m) TFA, CH_2_Cl_2_, 0 °C to rt, 1 h; n) L-phenylalanine benzyl ester DIEA, triphosgene, CH_2_Cl_2_, rt, 1 h, 60% over three steps; o) H_2_, Pd/C, EtOAc, rt, 2 h, 77%.

A straightforward route to the intriguing 2,3-pyrrolidinedione system appeared to be the Michael addition of a suitable amine to ethyl acrylate, followed by a Dieckmann cyclization with diethyl oxalate [10–11]. We chose the *p*-methoxybenzyl (PMB) protecting group for the amine, because of its facile cleavage with cerium ammonium nitrate (CAN) or 2,3-dichloro-5,6-dicyanobenzoquinone (DDQ). Thus, 2,3-pyrrolidinedione **3** was obtained by the reaction of ethyl acrylate with *p*-methoxybenzylamine, followed by treatment with diethyl oxalate (Scheme 1) [12]. On the basis of NMR data, the compound exists as an enol tautomer (see Supporting Information File 1). Indeed, perusal of the literature indicated that apparently all 4-monosubstituted 2,3-pyrrolidinediones are highly enolized [13–15]. Protection of the enolic OH with a benzyl group, using BnBr and K_2_CO_3_, gave compound **4**. The reduction of **4** with DIBAL-H gave the corresponding primary alcohol, which was converted into bromide **5** by Appel reaction with PPh_3_ and CBr_4_. The phosphonium salt obtained from this bromide was subjected to a Wittig reaction with nonanal, to afford compound **6** [12].

Attempts to remove the PMB protecting group (CAN, DDQ, TFA or hydrogenation) from **6** or from the intermediate alcohol and bromide resulted in the decomposition of the products. The instability of these and other derivatives of the N-unsubstituted ring, already observed by others [11], made the synthetic task more troublesome than expected.

The NH-free pyrrolidinedione scaffold was found to be stable only in the presence of the ester group in position 4. In fact, a deprotected compound **7** could only be obtained by treatment of **4** with CAN.

This result prompted us to install the amino acid fragment on compound **7** before constructing the chain in position 4, so that it could also act as a NH-protecting group in the following steps. However, as the ureido fragment would interfere with the reduction of the ester group on the nucleus, we opted for installing sequentially the valine, then the phenylalanine residue.

Thus, compound **7** was coupled with pentafluorophenyl ester-activated *N*-Boc-valine to obtain compound **8** (Scheme 1). Treatment with DIBAL-H led to the reduction of both the ester and the CO group of the valine moiety (**9**). Compound **9** was obtained as a 9:1 mixture of diastereomers, the syn (*S*,*S*) diastereomer being the major one. The selectivity of the reduction is in agreement with previous results and is consistent with the Cram model for a chelation controlled reduction [16–18]. Both OH groups of compound **9** were oxidized by PCC to obtain aldehyde **10**. The long carbon chain was incorporated into **10** by Wittig olefination with *n*-nonyltriphenylphosphonium bromide and *n*-BuLi at −78 °C (**11**). Deprotection and treatment with triphosgene and phenylalanine benzyl ester at rt allowed the formation of compound **13**. Finally, catalytic hydrogenation afforded the desired leopolic acid A (**1**), whose spectroscopic data completely matched with those reported in the literature [1] (see Supporting Information File 2 for NMR spectra).

**Scheme 2:**
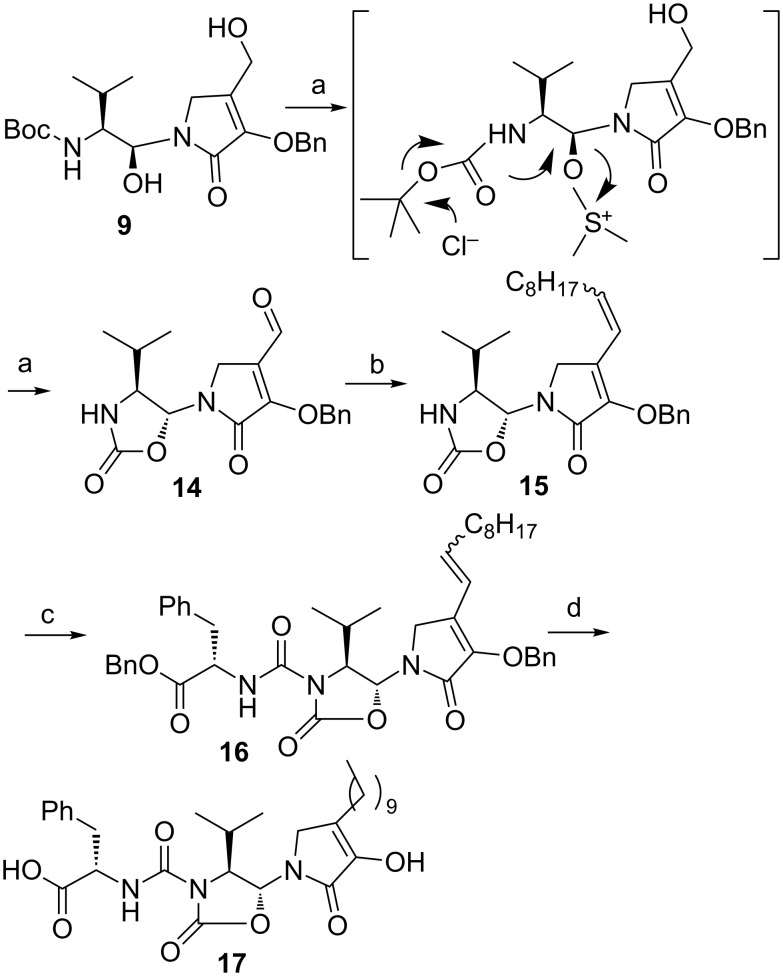
Synthesis of compound **17**. Reagents and conditions: a) Oxalyl chloride, DMSO, CH_2_Cl_2_, TEA, −78 °C to 0 °C, 2 h, 60%; b) *n*-nonyltriphenylphosphonium bromide, *n*-BuLi, THF, −78 °C to 0 °C, 2 h, 74%; c) L-phenylalanine benzyl ester, DIEA, triphosgene, CH_2_Cl_2_, rt, 72 h, 49%; d) H_2_, Pd/C, EtOAc, rt, 2 h, 50%.

Interestingly, while searching the best conditions to obtain compound **10**, we treated **9** with oxalyl chloride and DMSO, following the Swern protocol [19]. This treatment led to the formation of the oxazolidinone **14** (Scheme 2). We assume that the reaction occurred via inversion of the configuration at the hydroxylated carbon, based on an intramolecular S_N_2 cyclocarbamation by the Boc group [20–21]. The small coupling constant *(J* = 2.6 Hz) between the two hydrogens on the oxazolidinone ring confirmed that the compound was the *anti* derivative [16–17,22–23].

The oxazolidinone ring is rare among natural products, but there are successful examples in medicinal chemistry of drugs containing this skeleton, such as the antibiotic linezolid [24].

As the core structure of compound **14** represents a new scaffold, never isolated or synthesized before, we decided to incorporate the structural features found in leopolic acid into this novel heterocyclic system. Accordingly, Wittig olefination of **14**, followed by coupling with L-phenylalanine benzyl ester, and subsequent catalytic hydrogenation in EtOAc of **16** afforded the analogue of leopolic acid **17** in 50% yield.

Compounds **1** and **17** were subjected to a preliminary study of the antimicrobial activity against *Staphylococcus* and *Escherichia coli* (*Staphylococcus pseudintermedius* [25], 25 strains; *Escherichia coli*, 20 strains. QC: *S. aureus* ATCC 25923 and *Escherichia coli* ATCC 25922 were also used, see Supporting Information File 1 for details). The MIC values of **1** against *Staphylococcus pseudintermedius* range at 16 µg/mL, while the values for **17** against *Staphylococcus pseudintermedius* are more variable and range between 32 and 64 µg/mL. The MIC of **1** and **17** against *Escherichia coli* range at 128 µg/mL for both molecules (see Supporting Information File 2 for details). The antibacterial activity shown by both compounds suggests that they can be considered as promising candidates for further developments.

## Conclusion

In conclusion, leopolic acid A was obtained for the first time in a 11-step synthesis. The main difficulty encountered was the instability of a number of intermediates containing the 2,3-pyrrolidinedione moiety, which may be a reason for the scarce presence of similar compounds in the literature. With these results, we have attained a deeper knowledge of the chemistry of the unusual 2,3-pyrrolidinedione system and developed a synthetic strategy towards new lead compounds with antimicrobial activity. Efforts to synthesize analogues to build a structure–activity relationship (SAR) profile and optimize the activity are underway.

## Supporting Information

File 1 General experimental methods, synthetic procedures and analytical data for the reported compounds; antimicrobial activity evaluation procedures.

File 2 ^1^H and ^13^C NMR spectra of all the new compounds; 2D HMBC, HSQC spectra of compounds **1**, and **17**, COSY spectrum of compound **1**, MIC of compounds **1** and **17** against *Staphylococcus pseudintermedius* and *Escherichia coli* strains.
