# Supplementary material for: Total synthesis of leopolic acid A, a natural 2,3-pyrrolidinedione with antimicrobial activity
Source: Beilstein J Org Chem. 2016 Jul 29;12:1624–8. doi: 10.3762/bjoc.12.159 (PMC4979754; doi:10.3762/bjoc.12.159)
Supplement: File 1 — General experimental methods, synthetic procedures and analytical data for the reported compounds; antimicrobial activity evaluation procedures. [file Beilstein_J_Org_Chem-12-1624-s001.pdf]

Supporting Information- File 1

for

Total synthesis of leopolic acid A, a natural 2,3-pyrrolidinedione with antimicrobial activity

Atul A. Dhavan[1], Rahul D. Kaduskar[1], Loana Musso[1], Leonardo Scaglioni[1], Piera Anna Martino[2] and Sabrina Dallavalle[*1§]

Address: [1]Department of Food, Environmental and Nutritional Sciences, Division of Chemistry and Molecular Biology, Università degli Studi di Milano, via Celoria 2, I-20133 Milano, Italy and [2]Department of Veterinary Medicine - Microbiology and Immunology, Università degli Studi di Milano, via Celoria 10, I-20133 Milano, Italy

E-mail: Sabrina Dallavalle*- sabrina.dallavalle@unimi.it

[§]Tel. +39 0250316818; Fax +39 0250316801

* Corresponding author

General experimental methods, synthetic procedures and analytical data for the reported compounds; antimicrobial activity evaluation procedures

Experimental section

General information

All reagents and solvents were reagent grade or were purified by standard methods before use. Melting points were determined in open capillaries by a SMP3 apparatus and are uncorrected. ^{1}H and ^{13}C NMR spectra were recorded on Bruker AMX 300 MHz, Varian Mercury 300 MHz and Bruker AV600 spectrometers. TMS was used as an internal standard and the chemical shifts were reported in parts per million. The peak patterns are indicated as follows: s, singlet; d, doublet; dd, doublet of doublet; t, triplet; m, multiplet; q, quartet. The coupling constants, *J* are reported in Hertz (Hz). Optical rotations were measured with a Perkin Elmer 241 polarimeter. The elemental analyses were recorded with a CARLO ERBA EA 1108 instrument. Solvents were routinely distilled prior to use; anhydrous tetrahydrofuran (THF) and ether (Et_2O) were obtained by distillation from sodium-benzophenone ketyl; dry methylene chloride was obtained by distillation from phosphorus pentoxide. All reactions requiring anhydrous conditions were performed under a positive nitrogen flow and all glassware were oven dried and/or flame dried. Isolation and purification of the compounds were performed by flash column chromatography on silica gel 60 (230–400 mesh). Analytical thin-layer chromatography (TLC) was conducted on TLC plates (silica gel 60 F254, aluminum foil). Compounds on TLC plates were detected under UV light at 254 and 365 nm or were revealed spraying with 10% phosphomolybdic acid (PMA) in ethanol.

Compounds **3**,**5**,**6** were obtained following the procedures reported in the literature [1].

Ethyl 1-(4-methoxybenzyl)-4-(benzyloxy)-2,5-dihydro-5-oxo-1*H*-pyrrole-3-carboxylate (4). Anhydrous K_2CO_3 (21.45 g, 154.0 mmol) was added at 0 °C to a stirred solution of **3** [2] (15.02 g, 51.56 mmol) in dry DMF (150 mL) and the reaction was stirred for 15 min, then BnBr (9.52 g, 56.00 mmol) was added dropwise. The reaction mixture was allowed to warm to room temperature and was stirred for 1 h. After completion of the reaction, the mixture was diluted with EtOAc (150 mL) and washed with a cold brine solution (3 × 100 mL). The organic layer was dried over anhydrous Na_2SO_4, filtered and concentrated in vacuo. The crude product was purified by flash column chromatography (25% EtOAc/hexane) to afford **4** (10.03 g, 50%) as a white solid; mp 72 °C; R_f 0.5 (EtOAc/hexane 3:7); ^{1}H NMR ($CDCl_3$, 600 MHz) δ 7.54-7.45 (2H, m), 7.42-7.31 (3H, m), 7.18 (2H, d, *J* = 8.5 Hz), 6.88 (2H, d, *J* = 8.5 Hz), 5.82 (2H, s), 4.57 (2H, s), 4.24 (2H, q, *J* = 7.0 Hz), 3.86 (2H, s), 3.82 (3H, s), 1.30 (3H, t, *J* = 7.0 Hz); ^{13}C NMR ($CDCl_3$, 600 MHz) δ 164.9, 162.3, 159.3, 153.2, 136.7, 129.5 (× 2), 128.3 (× 2), 128.2, 128.0, 127.6 (× 2), 114.5, 114.2 (× 2), 72.5, 60.6, 55.2, 46.5, 46.2, 14.1; Anal. C 69.05, H 6.10, N 3.66%, calcd for $C_{22}H_{23}NO_5$, C 69.28, H 6.08, N 3.67.

Ethyl 4-(benzyloxy)-2,5-dihydro-5-oxo-1*H*-pyrrole-3-carboxylate (7). A stirred solution of **4** (5.00 g, 13.10 mmol) in CH_3CN:water (3:1) (40 mL) was added with cerium ammonium nitrate (28.74 g, 52.43 mmol) at 0 °C, then it was stirred for 3 h at room temperature. After the completion, the reaction mixture was diluted with EtOAc (100 mL) and washed with water (50 mL) and brine (60 mL) solution. The organic layer was dried over anhydrous Na_2SO_4, filtered and concentrated in vacuo. The crude product was purified by flash column chromatography (50% EtOAc/hexane) to afford **7** (2.50 g, 73%) as a white solid; mp 160 °C. R_f 0.3 (EtOAc/hexane 5:5) ^{1}H NMR ($CDCl_3$, 600 MHz) δ 7.82 (1H, brs), 7.51-7.42 (2H, m), 7.41-7.30 (3H, m), 5.77 (2H, s), 4.29 (2H, q, *J* = 7.0 Hz), 4.06 (2H, s), 1.34 (3H, t, *J* = 7.0 Hz); ^{13}C NMR

($CDCl_3$, 600 MHz) δ 168.4, 162.4, 153.1, 136.5, 128.4 (× 2), 128.1, 127.6 (× 2), 116.8, 72.9, 60.7, 43.0, 14.1; Anal. C 64.60, H 5.77, N 5.35%, calcd for $C_{14}H_{15}NO_4$, C 64.36, H 5.79, N 5.36.

(2*S*) 4-Benzyloxy-1-(2-*tert*-butoxycarbonylamino-3-methylbutyryl)-5-oxo-2,5-dihydro-1*H*-pyrrole-3-carboxylic acid ethyl ester (8). To a stirred solution of **7** (1.20 g, 4.59 mmol) in anhydrous THF (15 mL) was added *n*-BuLi (1.6 M in hexane, 2.6 mL, 4.17 mmol) at −78 °C over 10 min. After complete addition, the reaction mixture was stirred for 15 min. at −78 °C. To this a solution of 2-*tert*-butoxycarbonylamino-3-methyl-butyric acid pentafluorophenyl ester [3] (1.62 g, 4.25 mmol) in THF (2 mL) was added, and the mixture was stirred for 30 min at the same temperature. The reaction was quenched with sat. NH_4Cl (20 mL) and extracted with EtOAc (3 × 30 mL). The combined organic extracts were washed with brine (30 mL) dried over anhydrous Na_2SO_4, filtered and concentrated in vacuo. The crude product was purified by flash column chromatography (12% EtOAc/hexane) to afford **8** (1.50g, 71%) as a colorless viscous oil; $[\alpha]_D^{23}$ +11.2 (*c* 1.00, MeOH); R_f 0.5 (EtOAc/hexane 2:8); ^1H NMR ($CDCl_3$, 600 MHz) mixture of rotamers δ 7.50-7.31 (5H, m), 5.67 (2H, s), 5.41 (1H, m), 4.35 (1H, m), 4.31 (2H, q, J = 7.0 Hz); 4.30 (1H, m); 4.14 (2H, q, J = 7.0 Hz, 2nd rotamer), 2.15 (1H, m), 1.46 (9H, s), 1.34 (3H, t, J = 7.0 Hz), 1.28 (3H, t, J = 7.0 Hz, 2nd rotamer), 1.11 (3H, d, J = 7.0 Hz), 0.84 (3H, d, J = 7.0 Hz, 2nd rotamer). ^{13}C NMR ($CDCl_3$, 600 MHz) mixture of rotamers δ□(major): 172.5, 164.2, 161.7, 155.9, 150.8, 135.9, 128.6 (× 2), 128.5, 128.4 (× 2), 118.3, 79.8, 73.6, 61.3, 58.5, 44.6, 30.2, 28.2 (× 3), 19.8, 16.0, 14.1. Anal. C 62.80, H 7.02, N 6.09%, calcd for $C_{24}H_{32}N_2O_7$, C 62.59, H 7.00, N 6.08%.

(1*S*,1’*S*){1-[(3-Benzyloxy-4-(hydroxymethyl)-2-oxo-2,5-dihydro-1*H*-pyrrol-1-yl)-hydroxy-methyl]-2-methylpropyl}carbamic acid *tert*-butyl ester (9). A solution of **8** (1.00 g, 2.17 mmol) in dry CH_2Cl_2 (20 mL) was added with diisobutylaluminium hydride (1.0 M in CH_2Cl_2,

5.42 mL, 5.42 mmol) at −78 °C and stirred for 2 h at the same temperature. After completion of reaction, the mixture was quenched by addition of sat. Rochelle salt (20 mL) and EtOAc (40 mL) was added. The thick slurry obtained was stirred vigorously till the two layers became transparent. The organic phase was separated and the aqueous phase was extracted with EtOAc (3 × 25 mL). The combined organic extracts were washed with brine (35 mL) and dried over anhydrous Na_2SO_4, filtered and concentrated in vacuo. The crude product was purified by flash column chromatography (40% EtOAc/hexane) to afford **9** (0.27 g, 30%) as a yellowish solid; mp 60 °C; $[\alpha]_D^{23}$ -0.52 (*c* 1.00, MeOH); R_f 0.3 (EtOAc/hexane 4:6); ^{1}H NMR ($CDCl_3$, 300 MHz) δ 7.49-7.28 (5H, m), 5.44 (1H, dd, *J* = 4.6, 9.5 Hz), 5.25 (1H, d, *J* = 11.3 Hz), 5.11 (1H, d, *J* = 11.3 Hz), 4.62 (1H, d, *J* = 11.0 Hz), 4.46 (1H, d, *J* = 4.6 Hz), 4.31 (1H, d, J = 14.5 Hz), 4.12 (1H, d, J = 14.5 Hz), 4.04 (2H, s), 3.77 (2H, ddd, *J* = 3.5, 9.5, 11.0 Hz), 2.35 (1H, brs), 2.28-2.15 (1H, m), 1.33 (9H, s), 0.97 (3H, d, *J* = 6.7 Hz), 0.92 (3H, d, *J* = 6.7 Hz); ^{13}C NMR ($CDCl_3$, 300 MHz) δ 168.3, 156.6, 143.4, 136.8, 133.9, 128.8 (× 2), 128.6, 128.4 (× 2), 79.8, 75.9, 73.2, 56.9, 56.2, 43.9, 28.5 (× 2), 28.4, 28.0, 20.3, 15.6. Anal. C 62.93, H 7.65, N 6.67%, calcd for $C_{22}H_{32}N_2O_6$, C 62.84, H 7.67, N 6.66%.

(1*S*) [1-(3-Benzyloxy-4-formyl-2-oxo-2,5-dihydro-1*H*-pyrrole-1-carbonyl)-2-methyl-propyl]carbamic acid *tert*-butyl ester (10) To a stirred solution of compound **9** (0.22 g, 0.50 mmol) in CH_2Cl_2 (5 mL) PCC (1.12 g, 5.23 mmol) was added at 0 °C, then the solution was allowed to stir for 12 h at rt. After completion, the reaction mixture was filtered through a pad of celite and washed with CH_2Cl_2. The filtrate was concentrated in vacuo. The crude product was purified by flash column chromatography (20% EtOAc/hexane) to afford **10** (0.100 g, 46%) as a colourless viscous oil. $[\alpha]_D^{23}$ + 8.32 (*c* 1.00, MeOH); R_f 0.5 (EtOAc/hexane 2:8); ^{1}H NMR ($CDCl_3$, 300 MHz) δ 10.14 (1H, s), 7.51-7.35 (5H, m), 5.73-5.60 (2H, m), 5.39 (1H, dd, *J* = 2.8,

9.5 Hz), 5.17 (1H, d, *J* = 9.5 Hz), 4.39 (1H, d, *J* = 18.9 Hz); 4.24 (1H, d, J = 18.9 Hz), 2.19-2.05 (1H, m), 1.44 (9H, s), 1.09 (3H, d, *J* = 7.1 Hz), 0.84 (3H, d, J = 7.1 Hz); ^{13}C NMR ($CDCl_3$, 300 MHz) δ 185.3, 173.0, 164.6, 156.1, 153.8, 135.0, 129.3, 129.1, 128.4 (× 2), 125.0 (× 2), 80.1, 74.3, 58.7, 43.0, 30.5, 28.5 (× 3), 20.1, 16.3. Anal. C 62.56, H 6.75, N 6.71%, calcd for $C_{22}H_{28}N_2O_6$, C 63.45, H 6.78, N 6.73%.

(1*S*) [1-(3-Benzyloxy-4-dec-1-enyl-2-oxo-2,5-dihydro-1*H*-pyrrole-1-carbonyl)-2-methyl-propyl]-carbamic acid *tert*-butyl ester (11). To an ice cold solution of (1-nonyl)triphenylphosphonium bromide [4] (0.07 g, 0.14 mmol) in 3 mL of dry THF under nitrogen *n*-BuLi (1.6 M in hexane, 0.18 mL, 0.18 mmol) was added dropwise and the mixture was allowed stir for 45 min at the same temperature. The red solution was cooled to −78 °C and aldehyde **10** (0.03 g, 0.07 mmol) in anhydrous THF (2 mL) was added dropwise. The reaction was stirred for 1 h at −78 °C, warmed to 0 °C and quenched with sat. NH_4Cl (10 mL). The aqueous phase was extracted with EtOAc (2 × 15 mL). The combined organic extracts were washed with brine (10 mL) and dried over anhydrous Na_2SO_4, filtered and concentrated in vacuo to give crude **11** which was used for the next step without further purification. R_f 0.4 (EtOAc/hexane 1:9).

(1*S*) 1-(3-Benzyloxy-4-dec-1-enyl-2-oxo-2,5-dihydro-1*H*-pyrrole-1-carbonyl)-2-methyl-propylammonium trifluoroacetate (12). To a stirred solution of compound **11** (0.02 g, 0.04 mmol) in dry CH_2Cl_2 (1 mL) TFA (0.5 mL) was added at 0 °C. The reaction mixture was warmed to room temperature and stirred for 1 h. The reaction mixture was concentrated in vacuo. The residue was triturated with diethyl ether (3 × 5 mL) and dried to afford **12**. The crude product was used for the next step without further purification. R_f 0.2 (MeOH/CH_2Cl_2 1:9).

(2*S*,1'*S*) 2-{3-[1-(3-Benzyloxy-4-dec-1-enyl-2-oxo-2,5-dihydro-1*H*-pyrrole-1-carbonyl)-2-methylpropyl]ureido}-3-phenylpropionic acid benzyl ester (13). A mixture of L-phenylalanine benzyl ester (0.02 g, 0.04 mmol) and diisopropylethylamine (0.01 g, 0.08 mmol) in dry CH_2Cl_2 (2 mL) was added dropwise to a stirred solution of triphosgene (0.004 g, 0.013 mmol) in dry CH_2Cl_2 (2 mL) at rt. After 15 min stirring, a solution of **12** (0.03 g, 0.05 mmol) and DIEA (0.02 mL, 0.13 mmol) in dry CH_2Cl_2 (2 mL) was added dropwise. The reaction mixture was stirred for 1 h at rt, then the solvent was removed in vacuo. The residue was diluted with ethyl acetate (10 mL). The organic layer was washed with 10% aqueous $KHSO_4$ (5 mL), 5% aqueous $NaHCO_3$ (5 mL), brine (5 mL), dried over Na_2SO_4, filtered and concentrated in vacuo. The product was purified using PLC (35% EtOAc/hexane) to give **13** (31 mg, 60% over three steps) as a white viscous liquid. R_f 0.4 (EtOAc/hexane 3:7); ^{1}H NMR ($CDCl_3$, 300 MHz) (mixture of stereoisomers) δ 7.53-6.88 (15H, m), 6.36 (1H, d, J = 16.9 Hz), 6.15-5.97 (2H, m), 5.82-5.20 (4H, m), 5.16-4.77 (3H, m), 4.02 (1H, d, J = 19.1 Hz), 3.61 (1H, d, J = 19.1 Hz), 3.20-2.90 (2H, m), 2.21-2.07 (3H, m), 1.78-1.63 (2H, m), 1.52-0.64 (19H, m); ^{13}C NMR ($CDCl_3$, 300 MHz) (major stereoisomer) δ 174.0, 172.6, 165.7, 157.1, 141.3, 139.1, 137.0, 136.1, 135.4, 135.0, 129.7 (× 2), 128.8 (× 2), 128.75 (× 2), 128.7(× 2), 128.6 (× 2), 128.57 (× 2), 128.5 (× 2), 127.0, 119.5, 73.1, 67.1, 57.6, 53.8, 44.6, 39.3, 33.8, 32.1, 31.2, 29.6, 29.5, 29.46, 29.1, 22.9, 20.1, 16.6, 14.3. Anal. C 72.83, H 7.56, N 5.95%, calcd for $C_{43}H_{53}N_3O_6$, C 72.96, H 7.55, N 5.94%.

(2*S*,1'*S*) 2-{3-[1-(4-Decyl-3-hydroxy-2-oxo-2,5-dihydro-1*H*-pyrrole-1-carbonyl)-2-methyl-propyl]ureido}-3-phenylpropionic acid (1). To the solution of **13** (0.03 g, 0.04 mmol) in EtOAc (5 mL) was added a catalytic amount of 10% Pd/C (6 mg, 20% w/w). The reaction mixture was evacuated and flushed with H_2 gas (3 times), then stirred for 2 h at rt under H_2

atmosphere. The reaction mixture was filtered through a celite pad and the pad was washed with EtOAc (3 ×5 mL). The filtrate was concentrated in vacuo. The crude product was purified by reverse phase PLC (25% water/MeOH) to afford **1** (0.02 g, 77 %) as a clear oil; R_f 0.4 (H_2O/MeOH 2.5:7.5); ^{1}H NMR (DMSO-d_6, 600 MHz) δ: 12.62 (1H, s), 9.42 (1H, s), 7.27 (2H, dd, *J* = 7.6, 7.4 Hz), 7.21 (1H, dd, *J* = 7.6, 7.6 Hz), 7.19 (2H, d, *J* = 7.4 Hz), 6.47 (1H, d, *J* = 9.2 Hz), 6.34 (1H, d, *J* = 8.0 Hz), 5.37 (1H, dd, J = 9.2, 3.7 Hz); 4.28 (1H, m); 4.10 (1H, d, J = 18.0 Hz), 4.00 (1H, d, *J* = 18.0 Hz), 2.99 (1H, dd, *J* = 13.7, 5.1 Hz), 2.85 (1H, dd, *J* = 13.7, 7.5 Hz), 2.31 (2H, m), 2.00 (1H, m), 1.54-1.10 (16H, m), 0.91 (3H, d, *J* = 6.9 Hz); 0.86 (3H, t, *J* = 6.4 Hz); 0.76 (3H, d, *J* = 6.9 Hz); ^{13}C NMR (DMSO-d_6, 600 MHz) δ:174.2, 172.7, 166.5, 157.9, 141.4, 138.0, 129.8 (× 2), 129.1, 128.6 (× 2),126.8, 57.1, 54.6, 46.7, 38.0, 31.8, 30.3, 29.5 (× 2), 29.4 (× 2), 29.2, 27.3, 25.3, 22.6, 20.2, 16.8, 14.5. Anal. C 65.84, H 8.16, N 7.91%, calcd for $C_{29}H_{43}N_3O_6$, C 65.76, H 8.18, N 7.93%.

(4’*S*,5’*R*) 4-Benzyloxy-1-(4’-isopropyl-2’-oxooxazolidin-5’-yl)-5-oxo-2,5-dihydro-1*H*-pyrrole-3-carbaldehyde (14). The compound was prepared in a similar manner as described in ref. [5]. To a stirred solution of oxalyl chloride (0.37 mL, 4.30 mmol) in dry CH_2Cl_2 (3 mL), was added DMSO (0.4 mL, 5.72 mmol) in dry CH_2Cl_2 (3 mL) dropwise at −78 °C under N_2 atmosphere. The reaction mixture was stirred for 15 min, then **9** (0.60 g, 1.43 mmol) in dry CH_2Cl_2 (10 mL) was added dropwise. After complete addition, the reaction mixture was stirred at −78 °C for 1 h, then NEt_3 (1.98 mL, 14.33 mmol) was added dropwise. The reaction mixture was gradually warmed to 0 °C and stirred at this temperature till complete conversion was observed (2 h). The mixture was diluted with diethyl ether (10 mL) and poured in cold sat. $NaHCO_3$ (15 mL). The organic layer was separated; and the aqueous layer was extracted with diethyl ether (2 × 15 mL). The combined organic extracts were washed with brine (15 mL), dried

over anhydrous Na_2SO_4 and concentrated in vacuo. The crude product was purified by flash column chromatography (20% EtOAc/Hexane) to afford **14** (0.3 g, 60%) as a yellowish sticky solid. $[\alpha]_D^{23}$ = -0.90 (*c* , 1.00, MeOH), R_f 0.5 (EtOAc/Hexane 5:5) ^{1}H NMR ($CDCl_3$, 600 MHz) δ 10.11 (1H, s), 7.49-7.36 (5H, m), 6.92-6.75 (1H, m), 6.18 (1H, d, *J* = 3.9 Hz), 5.72 (2H, s), 4.19-4.02 (2H, m), 3.49 (1H, dd, *J* = 3.9, 6.7 Hz), 1.95-1.75 (1H, m), 1.01 (3H, d, *J* = 6.7 Hz)*;* 0.99 (3H, d, *J* = 6.7 Hz); ^{13}C NMR ($CDCl_3$, 600 MHz) δ 184.7, 165.2, 157.6, 154.7, 135.0, 128.9 (× 2); 128.8, 128.1 (× 2), 123.2, 81.2, 73.8, 61.7, 40.5, 32.1, 17.4 (× 2). Anal. C 62.86, H 5.84, N 8.11%, calcd for $C_{18}H_{20}N_2O_5$, C 62.78, H 5.85, N 8.13%.

(4'*S*,5'*R*) 5-(3-Benzyloxy-4-dec-1-enyl-2-oxo-2,5-dihydro-1*H*-pyrrol-1-yl)-4-isopropyl-oxazolidin-2-one (15). To a solution of (1-nonyl)triphenylphosphonium bromide (0.62 g, 1.32 mmol) in anhydrous THF (10 mL) was added dropwise *n*-BuLi (0.79 mL, 1.6 M, 1.26 mmol) at 0 °C under N_2 atmosphere. The resulting red solution was stirred for 45 min at 0 °C, then cooled to −78 °C. Aldehyde **14** (0.20 g, 0.63 mmol) in anhydrous THF (10 mL) was added dropwise. After complete addition, the reaction was stirred for 1 h at −78 °C, then warmed to 0 °C over 1 h. The reaction mixture was quenched with sat. NH_4Cl (20 mL). The aqueous phase was extracted with EtOAc (3 × 15 mL). The combined organic extracts were washed with brine (20 mL), dried over anhydrous Na_2SO_4, filtered and concentrated in vacuo. The crude product was purified by flash column chromatography (40% EtOAc/hexane) to afford **15** (0.205 g, 74%) as a white viscous oil. R_f 0.6 (EtOAc/hexane 5:5); $[\alpha]_D^{23}$ = -42.5 (*c* 0.35, MeOH), ^{1}H NMR ($CDCl_3$, 300 MHz) mixture of stereoisomers δ 7.44-7.28 (5H, m), 6.27-6.15 (2H, m), 5.94 (1H, brs), 5.65-5.55 (1H, m), 5.45-5.30 (2H, m), 4.16 (1H, d, *J* = 16.8 Hz), 4.04 (1H, d, *J* = 16.8 Hz), 3.52-3.40 (1H, m), 2.18-2.06 (2H, m), 1.89-1.64 (4H, m), 1.50-1.15 (12H, m), 1.02 (3H, d, J = 6.7 Hz), 0.97 (3H, d, J = 6.7 Hz); ^{13}C NMR ($CDCl_3$,, 600MHz) δ 166.6, 157.2, 143.5, 137.0, 136.9, 130.2,

128.4 (× 2), 128.2, 128.1 (× 2), 117.5, 81.6, 72.5, 61.4, 44.3, 32.1, 31.8, 29.9, 29.6, 29.5, 29.4, 29.3, 29.2, 22.6, 17.5, 14.0. Anal. C 71.31, H 8.41, N 6.17%, calcd for $C_{27}H_{38}N_2O_4$, C 71.33, H 8.43, N 6.16%.

(2*S*,4'*S*,5'*R*) 2-{[5-(3-Benzyloxy-4-dec-1-enyl-2-oxo-2,5-dihydro-1*H*-pyrrol-1-yl)-4-isopropyl-2-oxooxazolidine-3-carbonyl]amino}-3-phenylpropionic acid benzyl ester (16). A mixture of L-phenylalanine benzyl ester (0.06 g, 0.14 mmol) and diisopropylethylamine (0.06 mL, 0.35 mmol) in dry CH_2Cl_2 (2 mL) was added dropwise to a stirred solution of triphosgene (0.01 g, 0.05 mmol) at rt under N_2 atmosphere. After 15 min stirring, a solution of **15** (0.06 g, 0.13 mmol) and DIEA (0.06 mL, 0.35 mmol) in dry CH_2Cl_2 (2 mL) was added in one portion. The reaction mixture was stirred for 3 days at rt, the solvent was removed in vacuo and the residue was dissolved in EtOAc (15 mL). The organic phase was washed with 10% aqueous $KHSO_4$ (5 mL), 5% aqueous $NaHCO_3$ (5 mL), and brine (5 mL), dried over Na_2SO_4, filtered and concentrated to give **16** (0.05g, 49%) as a white viscous liquid. R_f 0.5 (EtOAc/Hexane 5:5); $[\alpha]_D^{23}$ = -0.35 (*c* 0.7, MeOH); ^{1}H NMR ($CDCl_3$, 300 MHz) mixture *E:Z* 4:6 δ 8.24 (1H, d, *J* = 7.8 Hz E + Z), 7.42-7.07 (15H, m, E + Z), 6.34 (1H, d, *J* = 16.2 Hz), 6.14 (1H, d, *J* = 11.8 Hz, Z), 6.12 (1H, d, *J* = 2.9 Hz, E), 6.06 (1H, d, *J* = 2.9 Hz, Z), 5.83 (1H, dt, *J* = 16.2, 6.9 Hz, E), 5.66 (1H, dt, *J* = 11.8, 7.2 Hz, Z), 5.37 (2H, s, Z), 5.36 (2H, s, E), 5.25-5.07 (2H, m, E + Z), 4.82-4.72 (1H, m, E + Z), 4.27 (1H, d, *J* = 2.9, 4.1 Hz, Z), 4.19 (1H, dd, *J* = 2.9, 4.1 Hz, E), 4.04-3.89 (2H, m, Z); 3.89-3.75 (2H, m, E); 3.27-3.05 (2H, m, E + Z), 2.53-2.29 (2H, m; E + Z); 2.22-1.96 (2H, m; E + Z); 1.84-1.53 (2H, m; E + Z); 1.45-1.11 (10H, m; E + Z); 0.98-0.79 (9H, m; E + Z). ^{13}CNMR ($CDCl_3$, 300 MHz) major isomer: δ 170.8, 166.8, 154.5, 150.8, 143.5, 137.8, 137.02, 135.9, 135.7, 135.3, 130.3, 129.5 (× 2), 128.9 (× 2), 128.8 (× 2), 128.7 (× 6), 128.4 (× 2),127.5, 117.7, 72.5, 67.5, 62.0, 54.9, 44.8, 38.1, 32.05, 30.3, 29.9, 29.7, 29.5, 29.4, 28.9, 22.9,

17.5, 15.0, 14.3. Anal. C 71.98, H 7.27, N 5.72%, calcd for $C_{44}H_{53}N_3O_7$, C 71.81, H 7.26, N 5.71%.

(2*S*,4'*S*,5'*R*) 2-{[5-(4-Decyl-3-hydroxy-2-oxo-2,5-dihydro-1*H*-pyrrol-1-yl)-4-isopropyl-2-oxo-oxazolidine-3-carbonyl]amino}-3-phenylpropionic acid (17). To the solution of **16** (0.04 g, 0.05 mmol) in EtOAc (7 mL) was added 10% Pd/C (8 mg, 20% w/w). The reaction mixture was evacuated and flushed with hydrogen gas (3 times) and stirred at room temperature under hydrogen atmosphere for 2 h. After completion, the reaction mixture was filtered through a short pad of celite and the pad was washed with EtOAc (3 × 5 mL). The filtrate was concentrated *in vacuo*. The crude product was purified by preparative PLC (2% AcOH/CH_2Cl_2) to afford **17** (0.015 g, 50%) as a white viscous liquid; R_f 0.5 (MeOH/DCM 1:9); $[\alpha]_D^{23}$ -32 (*c* 1.00, MeOH); ^{1}H NMR (DMSO-d_6, 600 MHz) δ 9.4 (1H, brs), 8.17 (1H, d, *J* = 7.1 Hz), 7.24-7.13 (5H, m), 5.94 (1H, d, *J* = 2.6 Hz), 4.42 (1H, dd, *J* = 2.6, 3.5 Hz), 4.36-4.30 (1H, m), 3.96 (1H, d, *J* = 17.1 Hz, AB), 3.59 (1H, d, *J* = 17.1 Hz, AB), 3.15 (1H, dd, J = 3.4, 13.1 Hz), 3.04 (1H, dd, *J* = 6.0, 13.1 Hz), 2.40-2.26 (3H, m), 1.49-1.41 (2H, m), 1.31-1.18 (14H, m), 0.87 (3H, d, *J* = 6.9 Hz), 0.86-0.80 (6H, m); ^{13}C NMR (DMSO-d_6, 600 MHz) δ 172.0, 167.4, 153.8, 149.4, 140.9, 137.6, 129.4, 127.8, 126.1, 125.7, 77.1, 60.6, 55.0, 44.3, 37.0, 31.2, 29.0 (× 2), 28.9, 28.7, 28.6, 28.3, 27.3, 24.8, 22.0, 16.8, 14.5, 13.9. Anal. C 64.52, H 7.75, N 7.54%, calcd for $C_{30}H_{43}N_3O_7$, C 64.61, H 7.77, N 7.53%.

MIC determination

MIC were determined for each bacterial strain (*Staphylococcus* species and *Escherichia coli*) using the broth microdilution method, according to the Clinical and Laboratory Standards Institute (CLSI) reference methods [6]. The drug solutions were prepared in DMSO to obtain a

initial concentration of 1 mg/mL. Overnight cultures were prepared and adjusted to a density of 0.5 Mc Farland scale; Twentyfive strains of *Staphylococcus pseudintermedius* and twenty strains of *Escherichia coli* both from animal infections were used. QC: *S. aureus* ATCC 25923 and *Escherichia coli* ATCC 25922 were also used. For the tests 96-wells sterile microtiter plates were used and compounds dilutions (1:2) were performed in Mueller Hinton Broth (Thermo Fisher Scientific, Italy). Negative and positive control experiments were included. After incubation at 37 °C for 24 h, the MIC was determined as the lowest concentration of compound at which there was visible growth.

References

1. Dhavan, A. A.; Ionescu, A. C.; Kaduskar, R. D.; Brambilla, E.; Dallavalle, S. *Bioorg. Med. Chem. Lett.* **2016**, *26*, 1376–1380.

2. a) Sundberg, R.; Richard, J.; Bradley, C.; Laurino, J. P. *J. Heterocycl. Chem.* **1986**, *23*, 537-539; b) Coumar, M. S.; Wu, J. S.; Leou, J. S.; Tan, U. K.; Chang, C. Y.; Chang, T. Y.; Lin, W. H.; Hsu, J. T. A.; Chao, Y. S.; Wu, S. Y.; Hsieh, H. P. *Bioorg. Med. Chem. Lett.* **2008**, *18*, 1623-1627.

3.Ramapanicker, R.; Baig, N. B. R.; De, K.; Chandrasekaran, S. *Journal of Peptide Science*, **2009**, *15*, 849-855.

4. Li, G.; Watson, K.; Buckheit, R. W.; Zhang Y. *Organic Letters* **2007**, *9*, 2043-2046.

5. Kaduskar, R. D.; Dhavan, A. A.; Dallavalle, S; Scaglioni, L.; Musso, L. *Tetrahedron* **2016**, *72*, 2034-2041.

6. Clinical and Laboratory Standards Institute. Methods for dilution antimicrobial susceptibility tests for bacteria that grow aerobically; approved standard – tenth edition. CLSI document M07-A10, Clinical and Laboratory Standards Institute, Wayne, PA. 2015
