# Supplementary material for: Total synthesis of leopolic acid A, a natural 2,3-pyrrolidinedione with antimicrobial activity
Source: Beilstein J Org Chem. 2016 Jul 29;12:1624–8. doi: 10.3762/bjoc.12.159 (PMC4979754; doi:10.3762/bjoc.12.159)
Supplement: File 2 — 1H and 13C NMR spectra of all the new compounds; 2D HMBC, HSQC spectra of compounds 1, and 17, COSY spectrum of compound 1, MIC of compounds 1 and 17 against Staphylococcus pseudintermedius and Escherichia coli strains. [file Beilstein_J_Org_Chem-12-1624-s002.pdf]

Supporting Information- File 2

for

Total synthesis of leopolic acid A, a natural 2,3-pyrrolidinedione with antimicrobial activity

Atul A. Dhavan[1], Rahul D. Kaduskar[1], Loana Musso[1], Leonardo Scaglioni[1], Piera Anna Martino[2] and Sabrina Dallavalle[*1§]

Address: [1]Department of Food, Environmental and Nutritional Sciences, Division of Chemistry and Molecular Biology, Università degli Studi di Milano, via Celoria 2, I-20133 Milano, Italy and [2]Department of Veterinary Medicine - Microbiology and Immunology, Università degli Studi di Milano, via Celoria 10, I-20133 Milano, Italy

E-mail: Sabrina Dallavalle*- sabrina.dallavalle@unimi.it

[§]Tel. +39 0250316818; Fax +39 0250316801

* Corresponding author

^{1}H and ^{13}C NMR spectra of all the new compounds; 2D HMBC, HSQC spectra of compounds 1, and 17, COSY spectrum of compound 1, MIC of compounds 1 and 17 against *Staphylococcus pseudintermedius* and *Escherichia coli* strains

Table of contents

^{1}H-NMR Spectrum of compound **4** S3

^{13}C-NMR Spectrum of compound **4** S4

^{1}H-NMR Spectrum of compound **7** S5

^{13}C-NMR Spectrum of compound **7** S6
^{1}H-NMR Spectrum of compound **8** S7
^{13}C-NMR Spectrum of compound **8** S8
^{1}H-NMR Spectrum of compound **9** S9
^{13}C-NMR Spectrum of compound **9** S10
^{1}H-NMR Spectrum of compound **10** S11
^{13}C-NMR Spectrum of compound **10** S12
^{1}H-NMR Spectrum of compound **13** S13
^{13}C-NMR Spectrum of compound **13** S14
^{13}C-NMR Spectrum of compound **13** (exp.) S15
^{13}C-NMR Spectrum of compound **14** S16
^{1}H-NMR Spectrum of compound **14** S17
^{13}C-NMR Spectrum of compound **15** S18
^{1}H-NMR Spectrum of compound **15** S19
^{13}C-NMR Spectrum of compound **16** S20
^{13}C-NMR Spectrum of compound **16** S21
^{13}C-NMR Spectrum of compound **16** (exp) S22
^{1}H-NMR Spectrum of compound **17** S23
^{13}C-NMR Spectrum of compound **17** S24
COSY Spectrum of compound **17** S25
HSQC Spectrum of compound **17** S26
HMBC Spectrum of compound **17** S27
^{1}H-NMR Spectrum of compound **1** S28
^{13}C-NMR Spectrum of compound **1** S29
HSQC Spectrum of compound **1** S30
HMBC Spectrum of compound **1** S31
DEPT Spectrum of compound **1** S32
MIC of **1** against *Staphylococcus* strains S33
MIC of **1** against *E. Coli* strains S34
MIC of **17** against *Staphylococcus* strains S35
MIC of **17** against *E. Coli* strains S36

^{1}H NMR (600 MHz, $CDCl_3$) spectrum of compound **4**

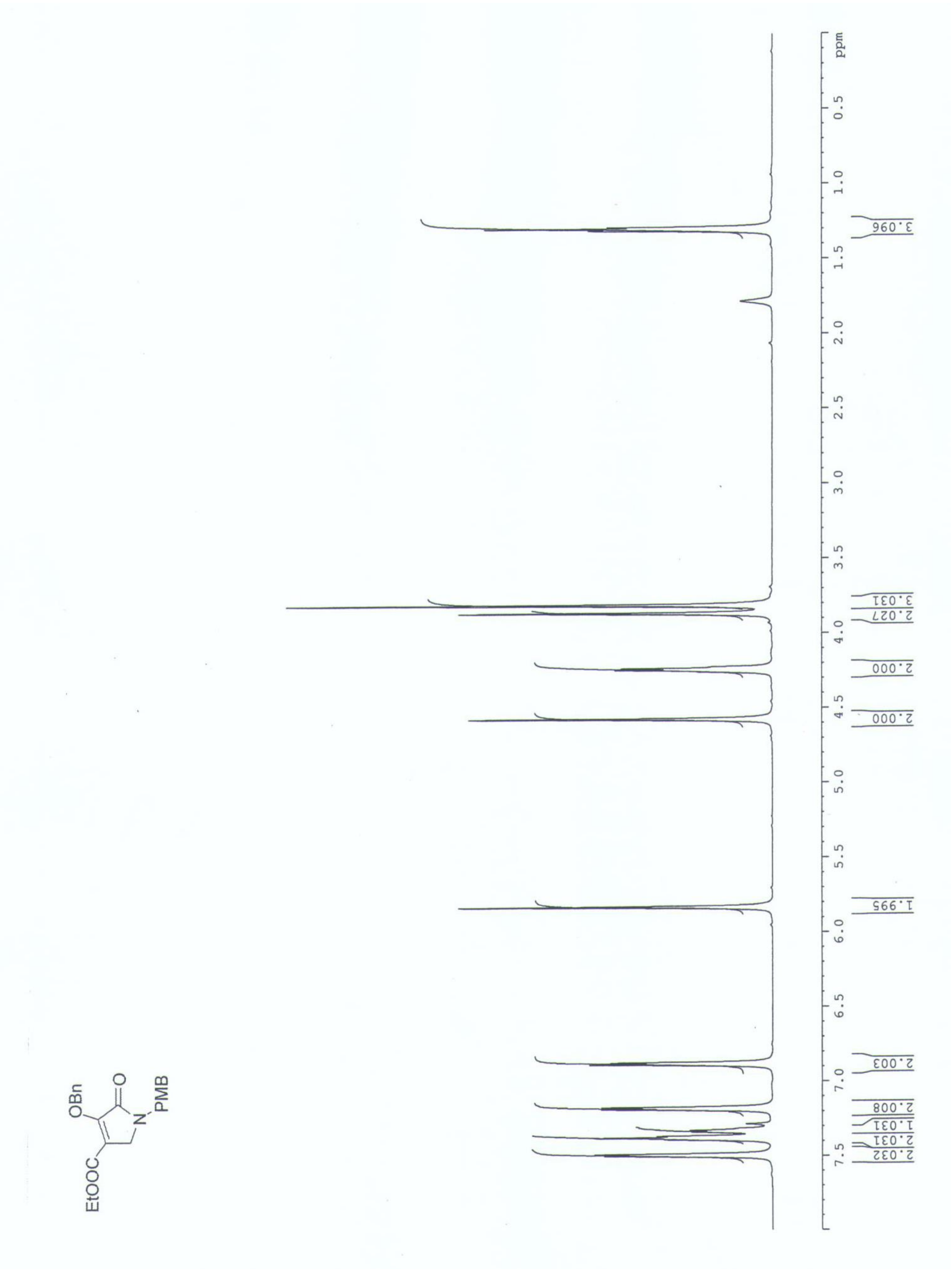

^{13}HC NMR (600 MHz, $CDCl_3$) spectrum of compound **4**

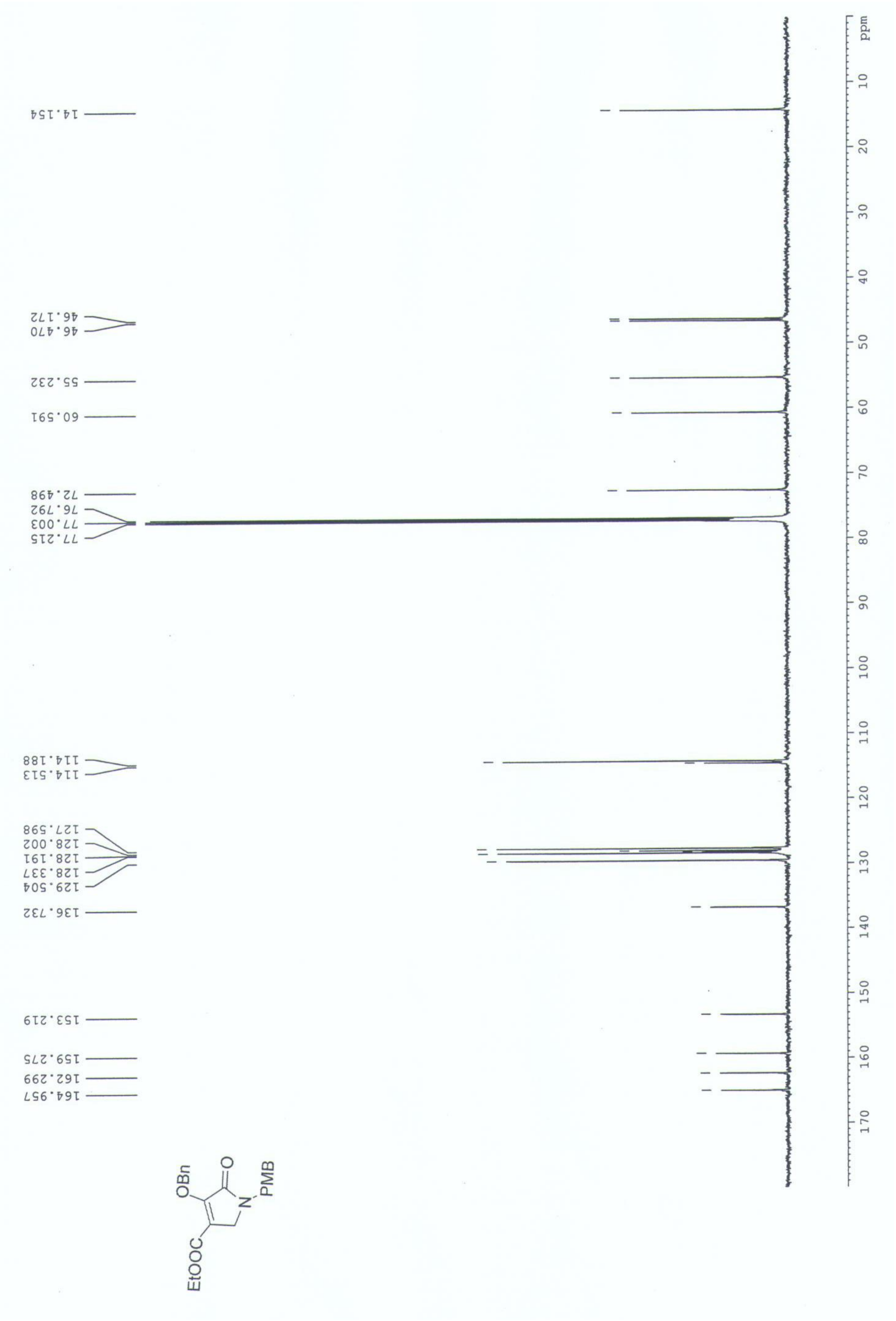

^{1}H NMR (600 MHz, $CDCl_3$) spectrum of compound **7**

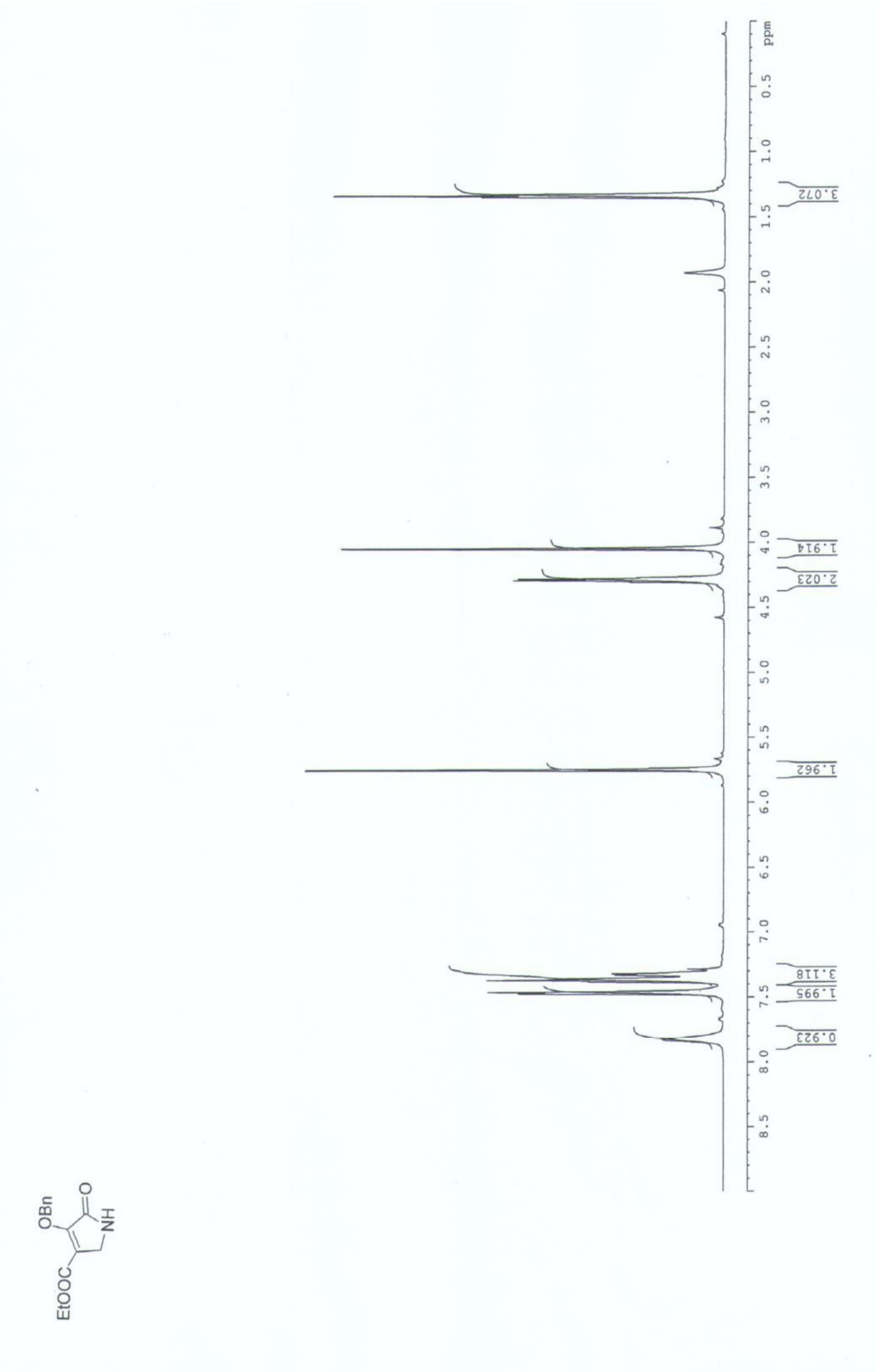

^{13}H NMR (600 MHz, $CDCl_3$) spectrum of compound **7**

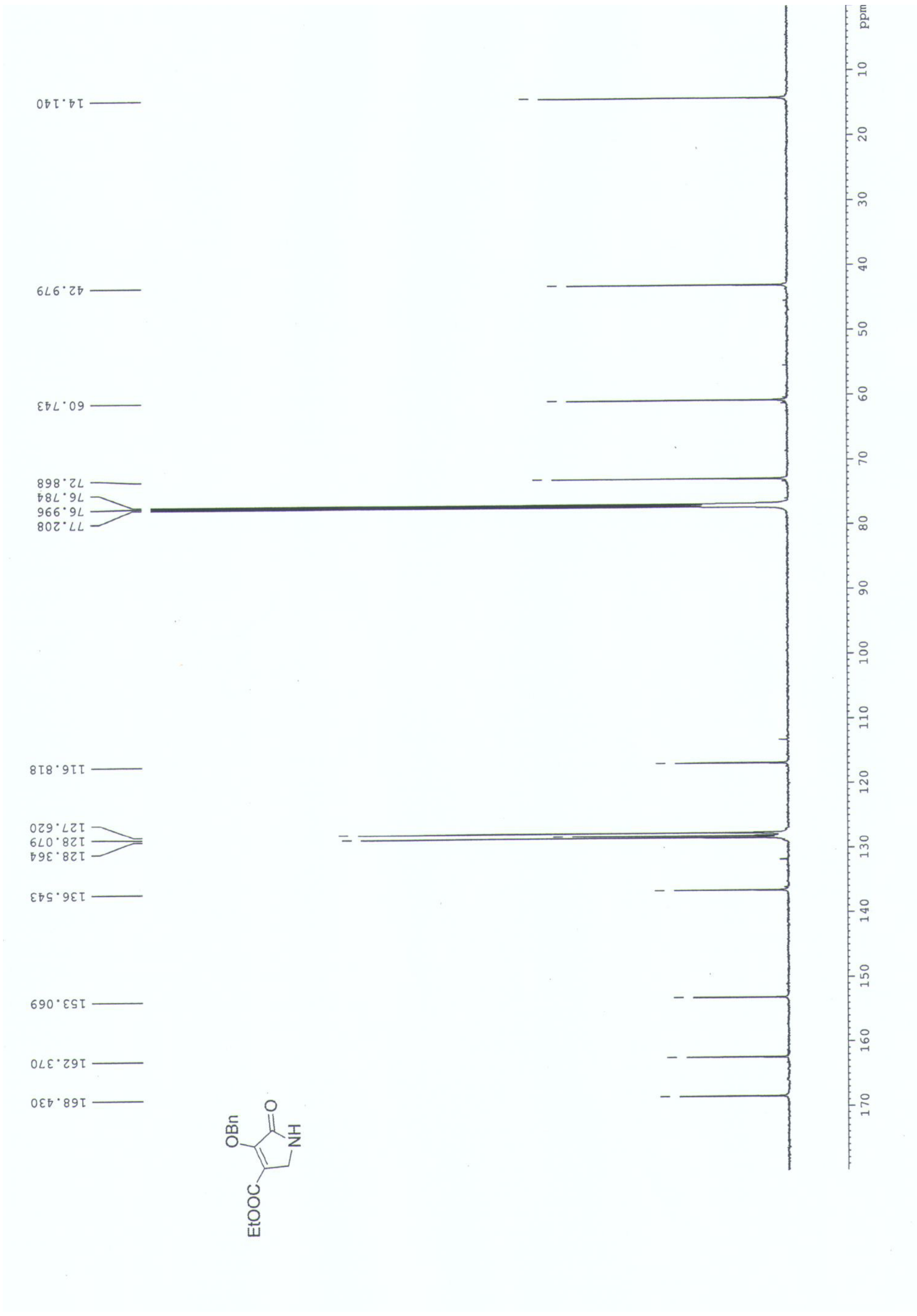

^{1}H NMR (600 MHz, $CDCl_3$) spectrum of compound **8** (mixture of rotamers)

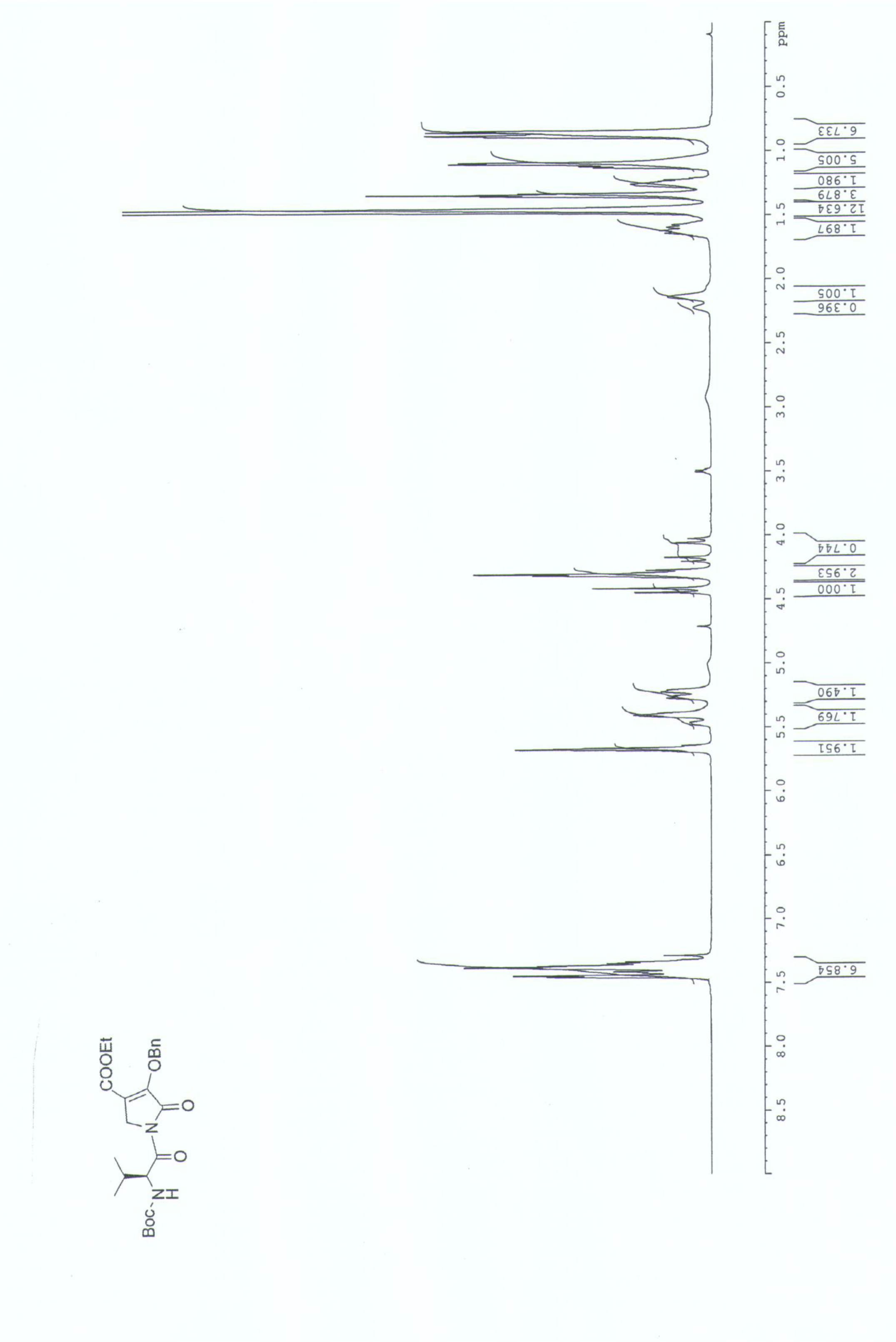

^{13}C NMR (600 MHz, $CDCl_3$) spectrum of compound **8** (mixture of rotamers)

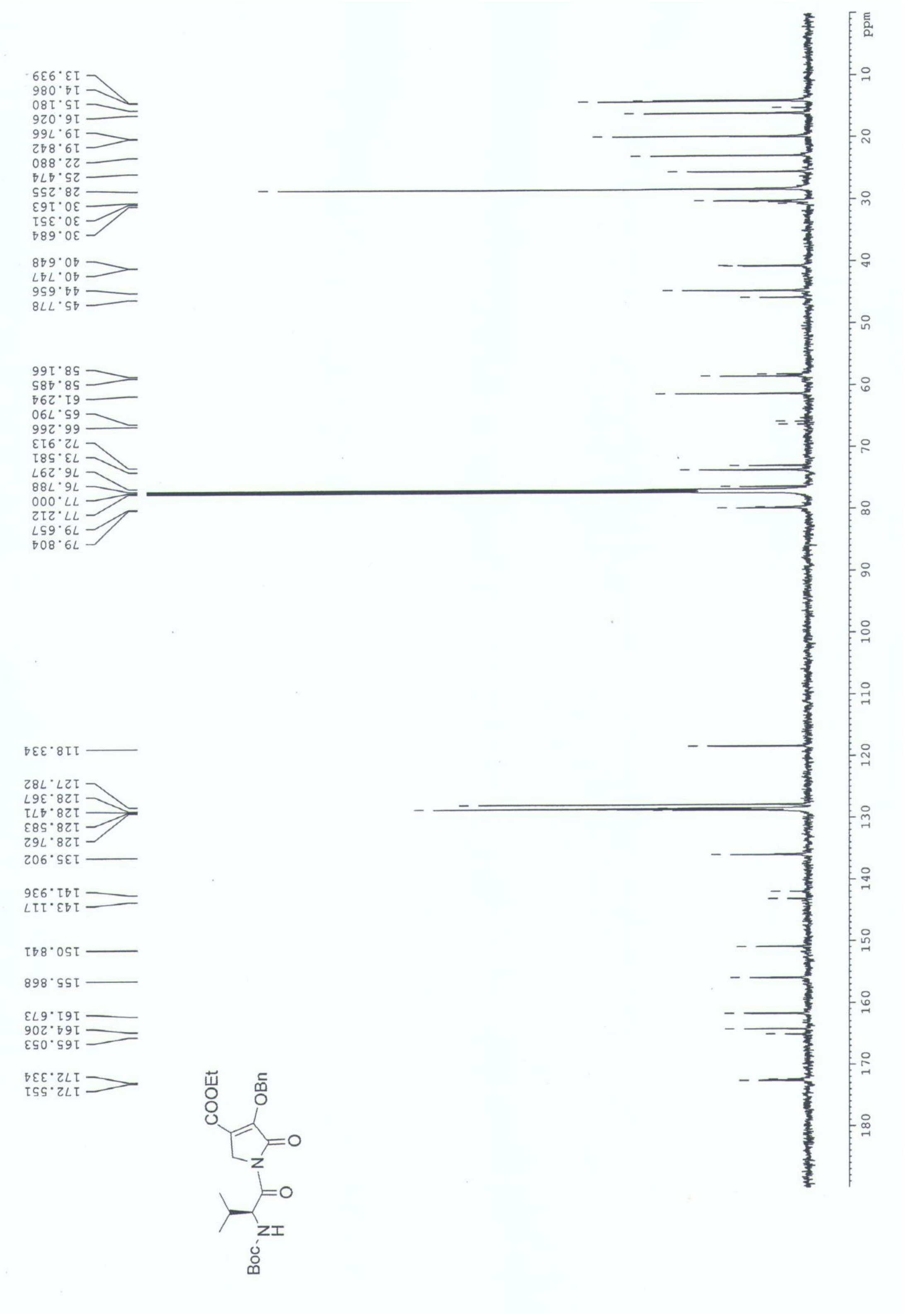

^{1}H NMR (300 MHz, $CDCl_3$) spectrum of compound **9**

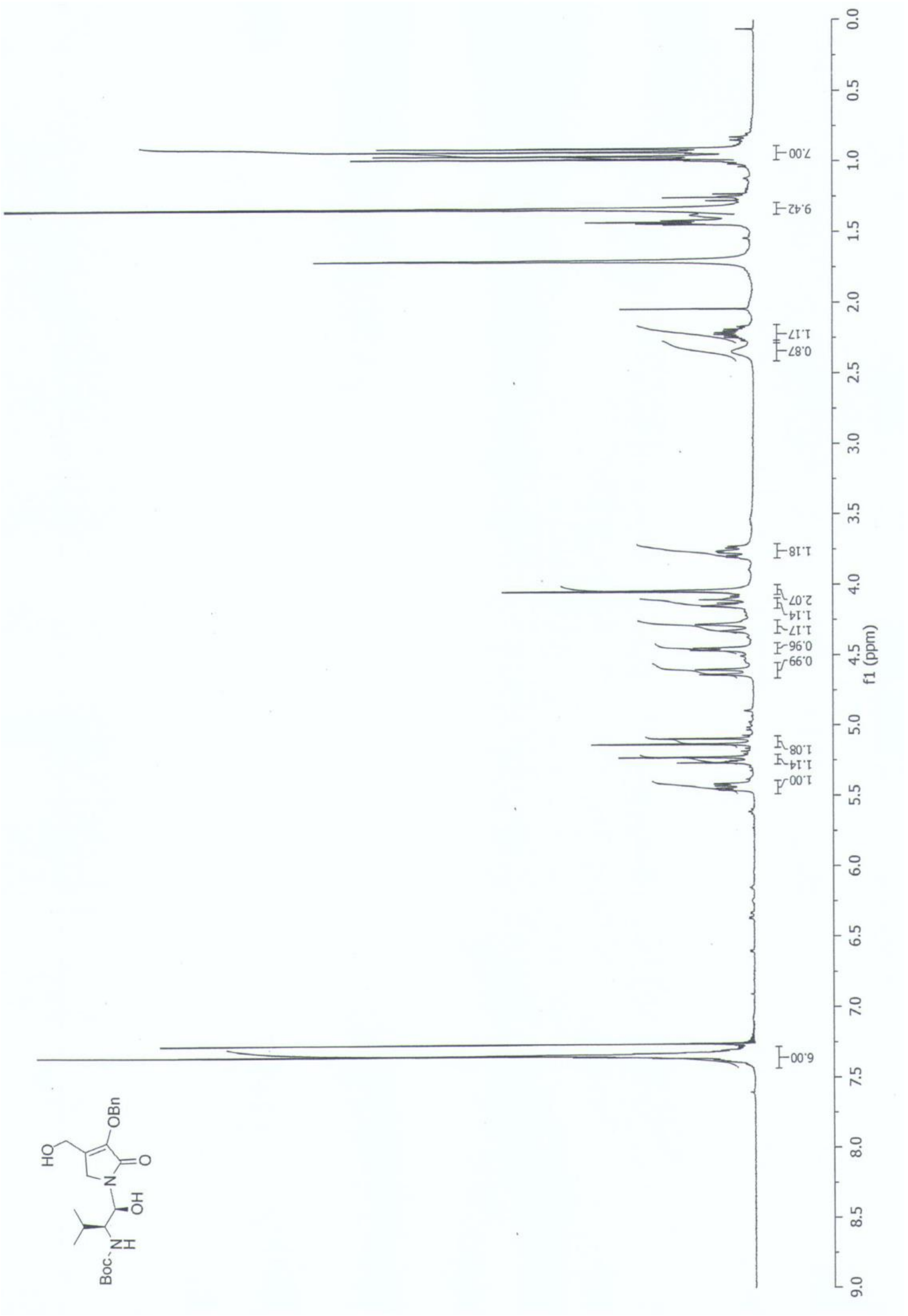

^{13}C NMR (300 MHz, $CDCl_3$) spectrum of compound **9** (APT)

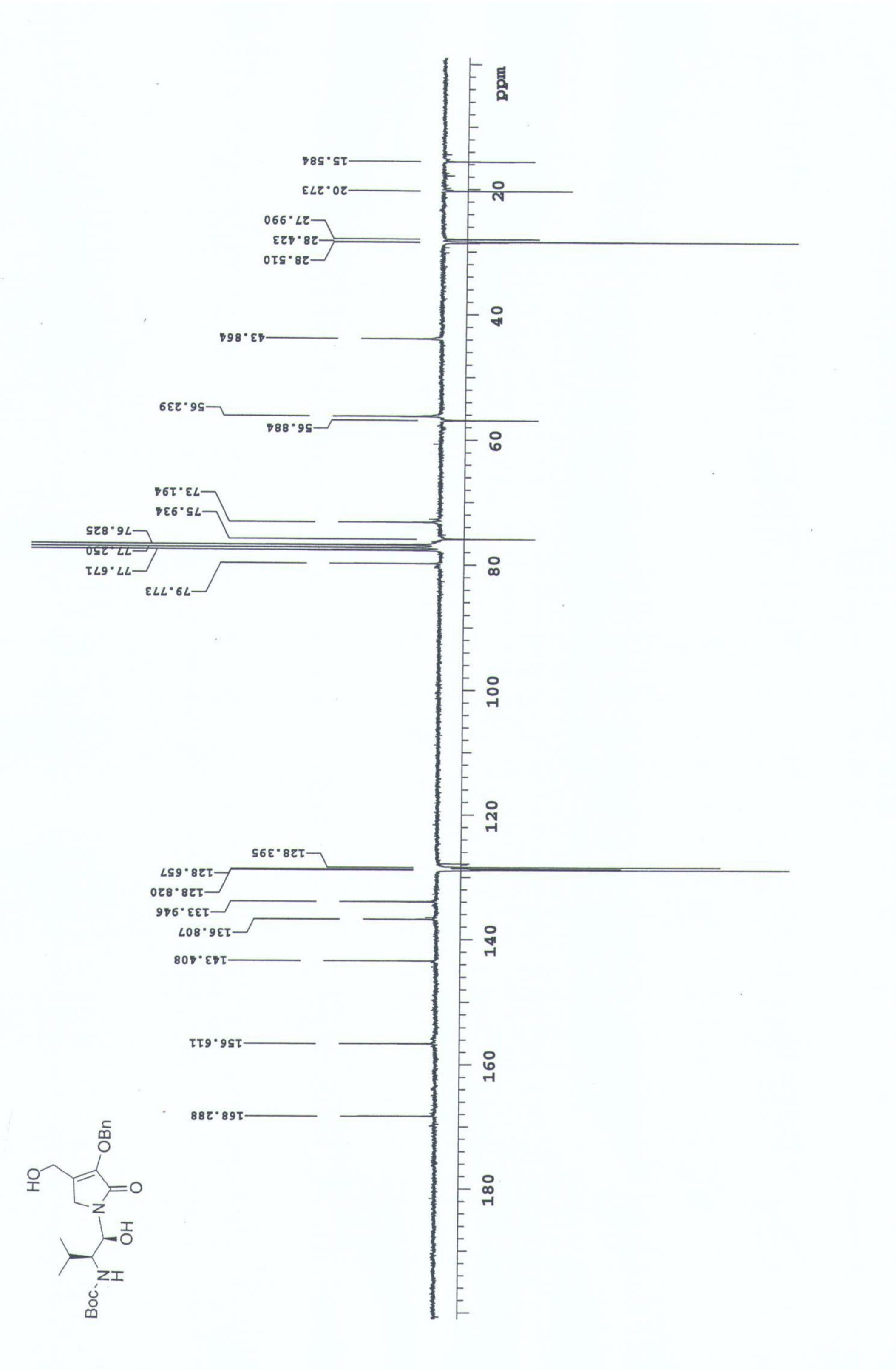

^{1}H NMR (300 MHz, $CDCl_3$) spectrum of compound **10**

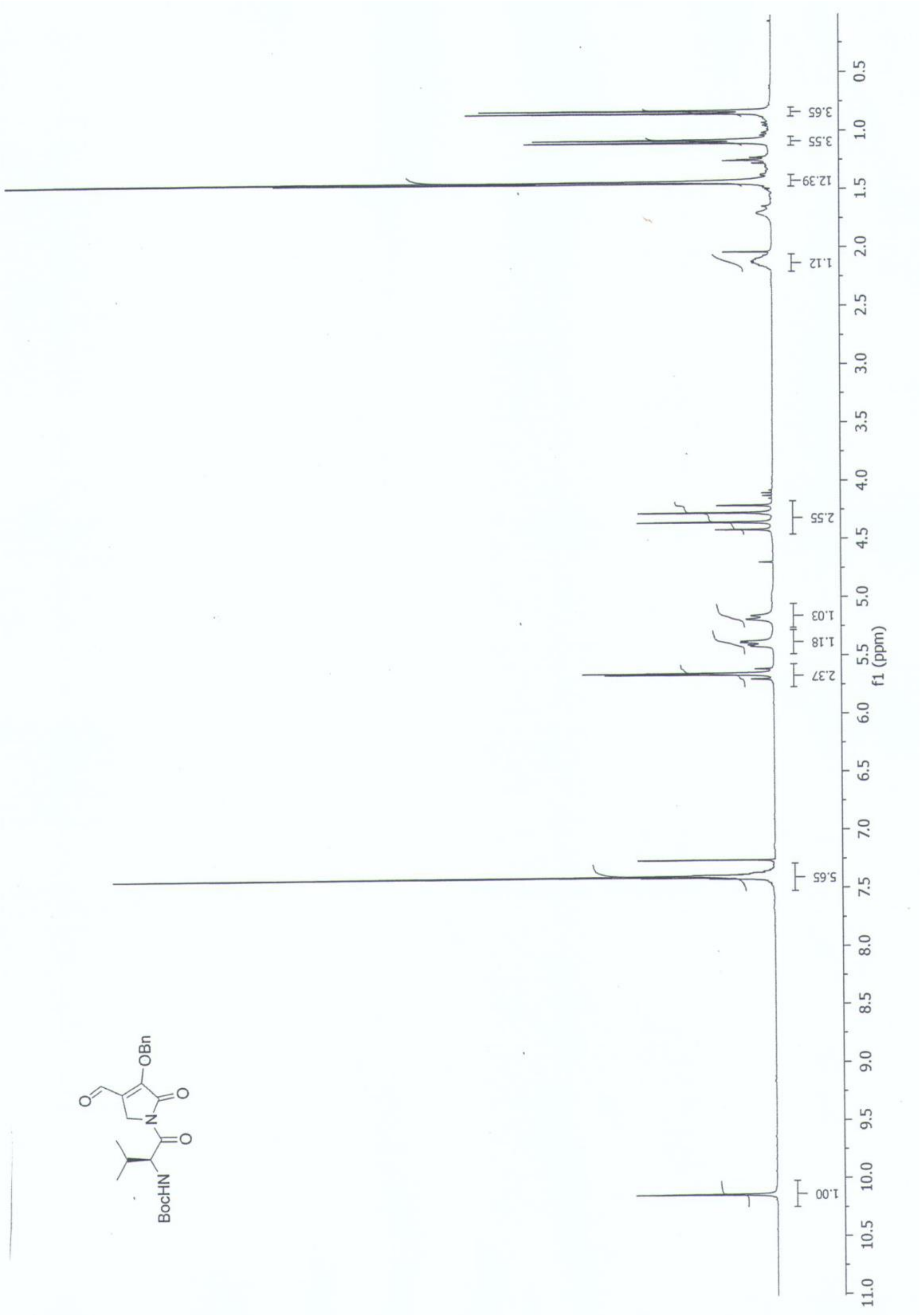

^{13}C NMR (300 MHz, $CDCl_3$) spectrum of compound **10** (APT)

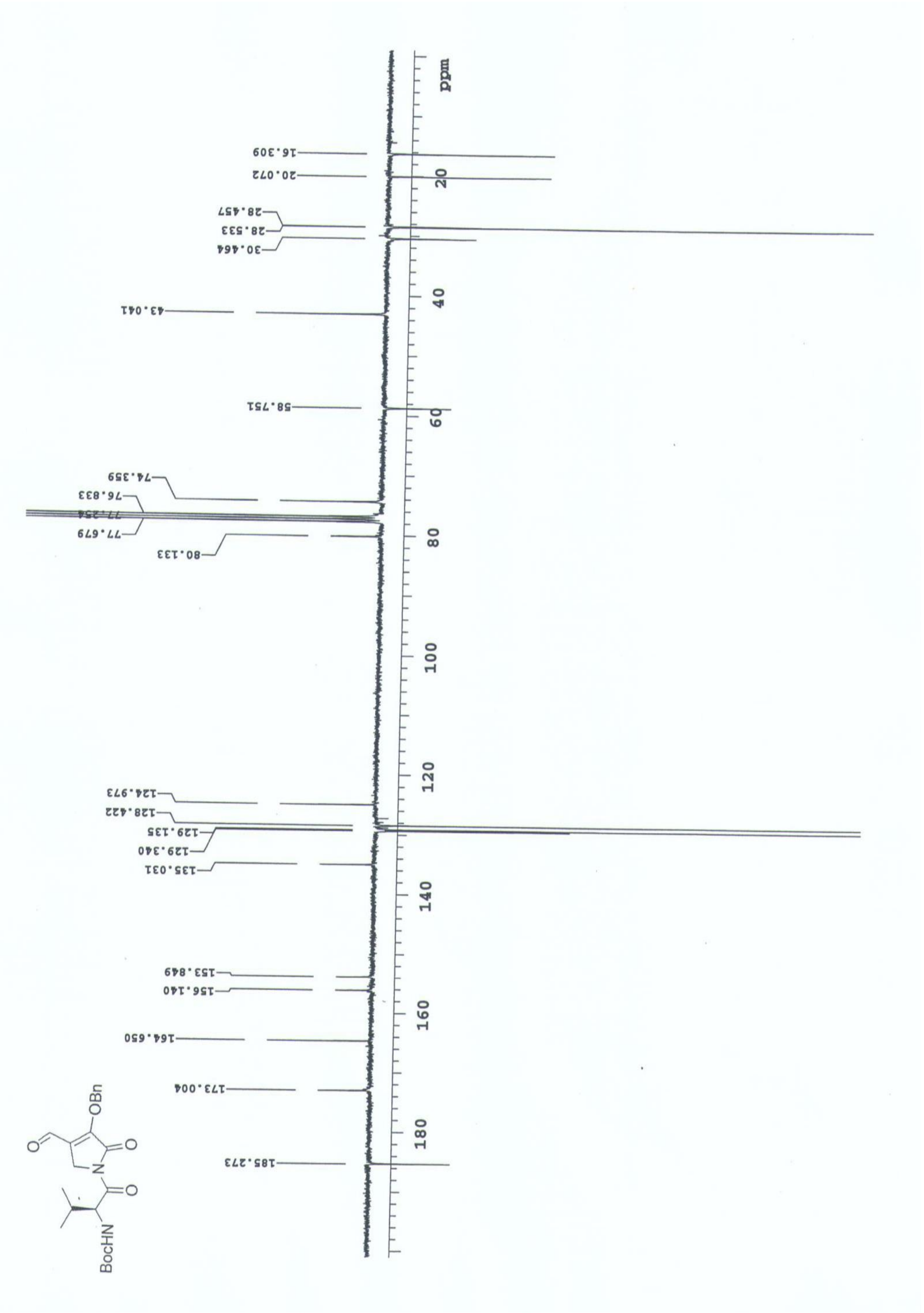

^{1}H NMR (300 MHz, $CDCl_3$) spectrum of compound **13** (mixture of stereoisomers)

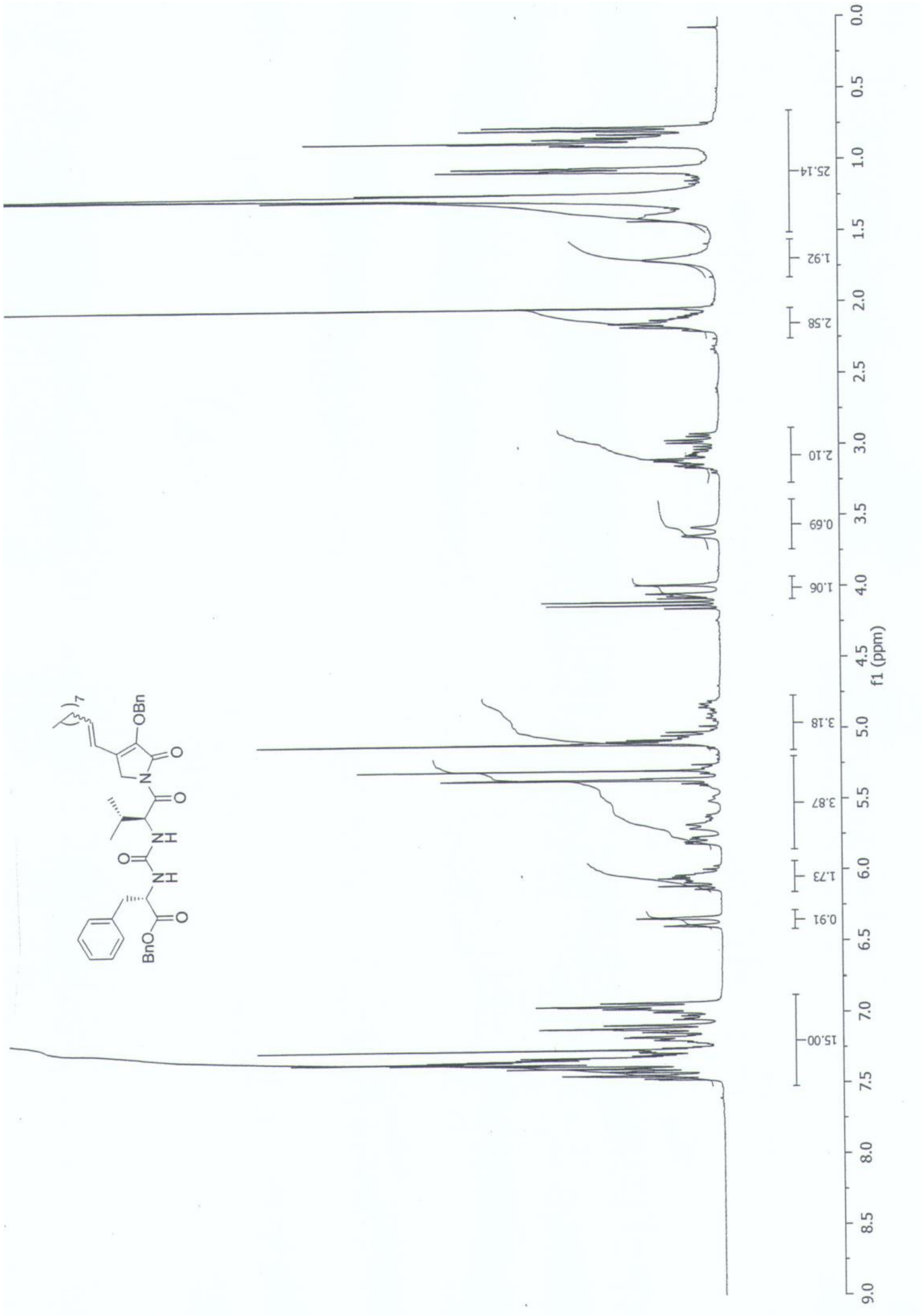

^{13}C NMR (300 MHz, $CDCl_3$) spectrum of compound **13** (mixture of stereoisomers) (APT)

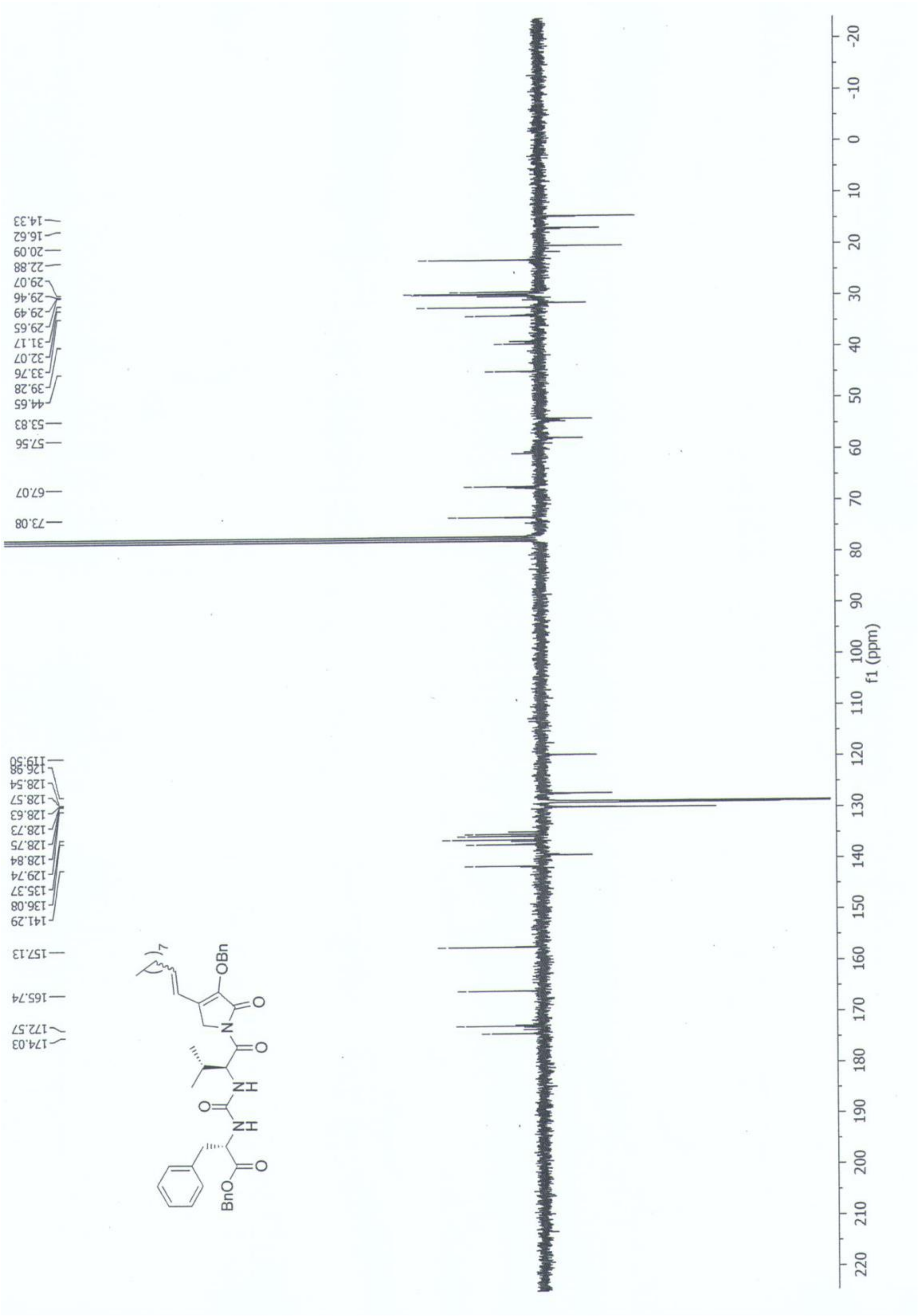

^{13}C NMR (300 MHz, CDCl3) spectrum of compound **13** (mixture of stereoisomers) (APT, expanded))

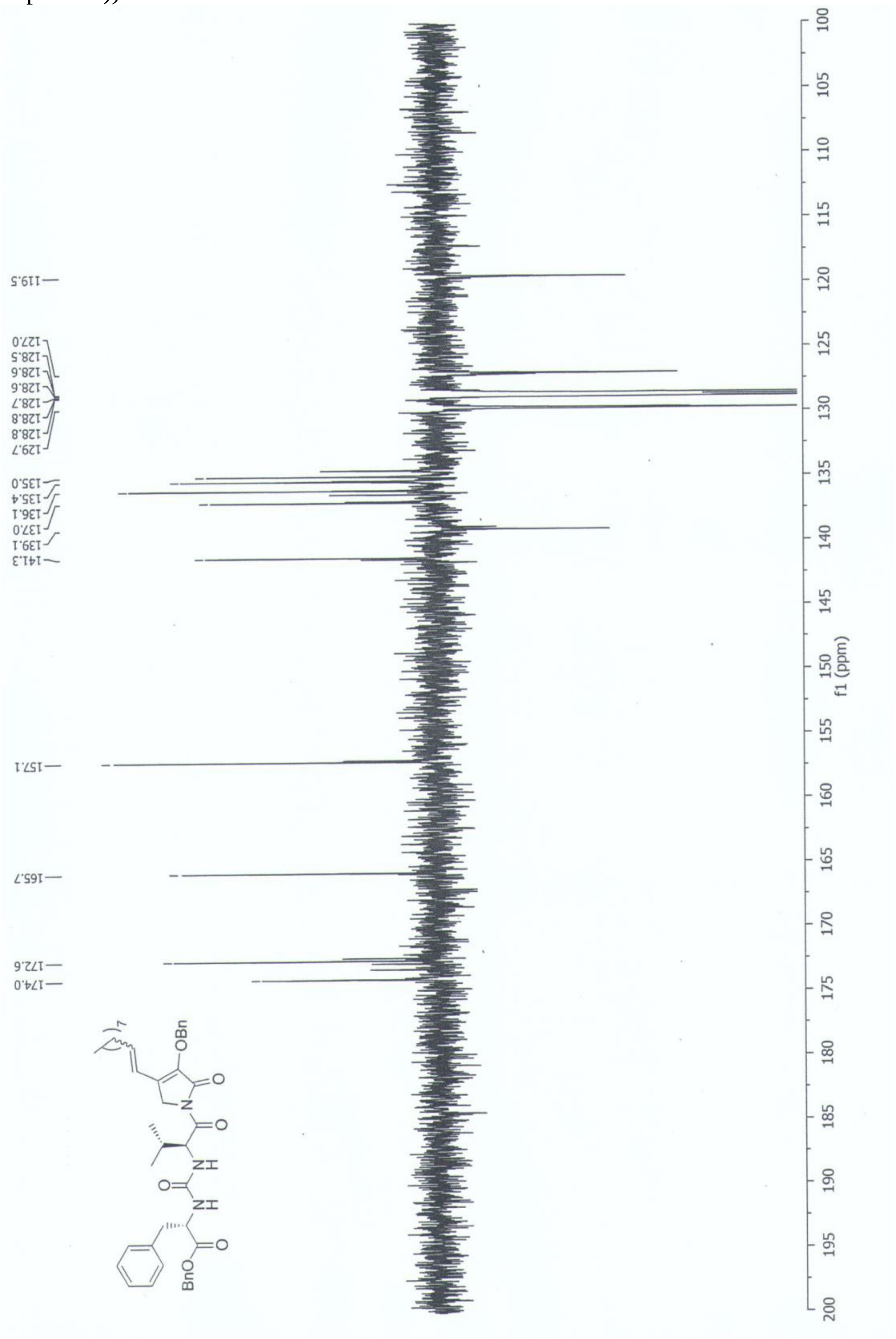

^{1}H NMR (600 MHz, CDCl3) spectrum of compound **14**

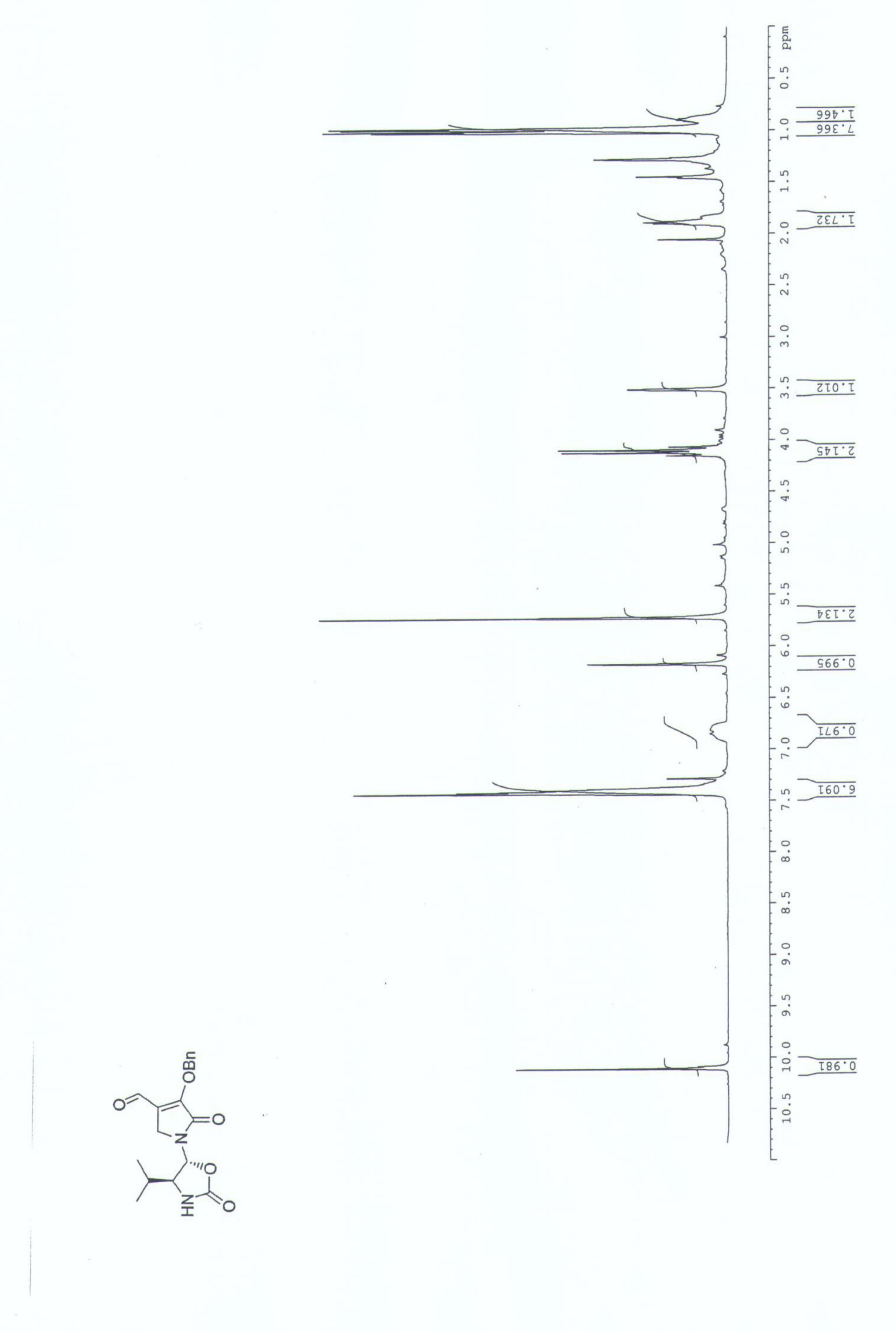

^{13}C NMR (600 MHz, $CDCl_3$) spectrum of compound **14**

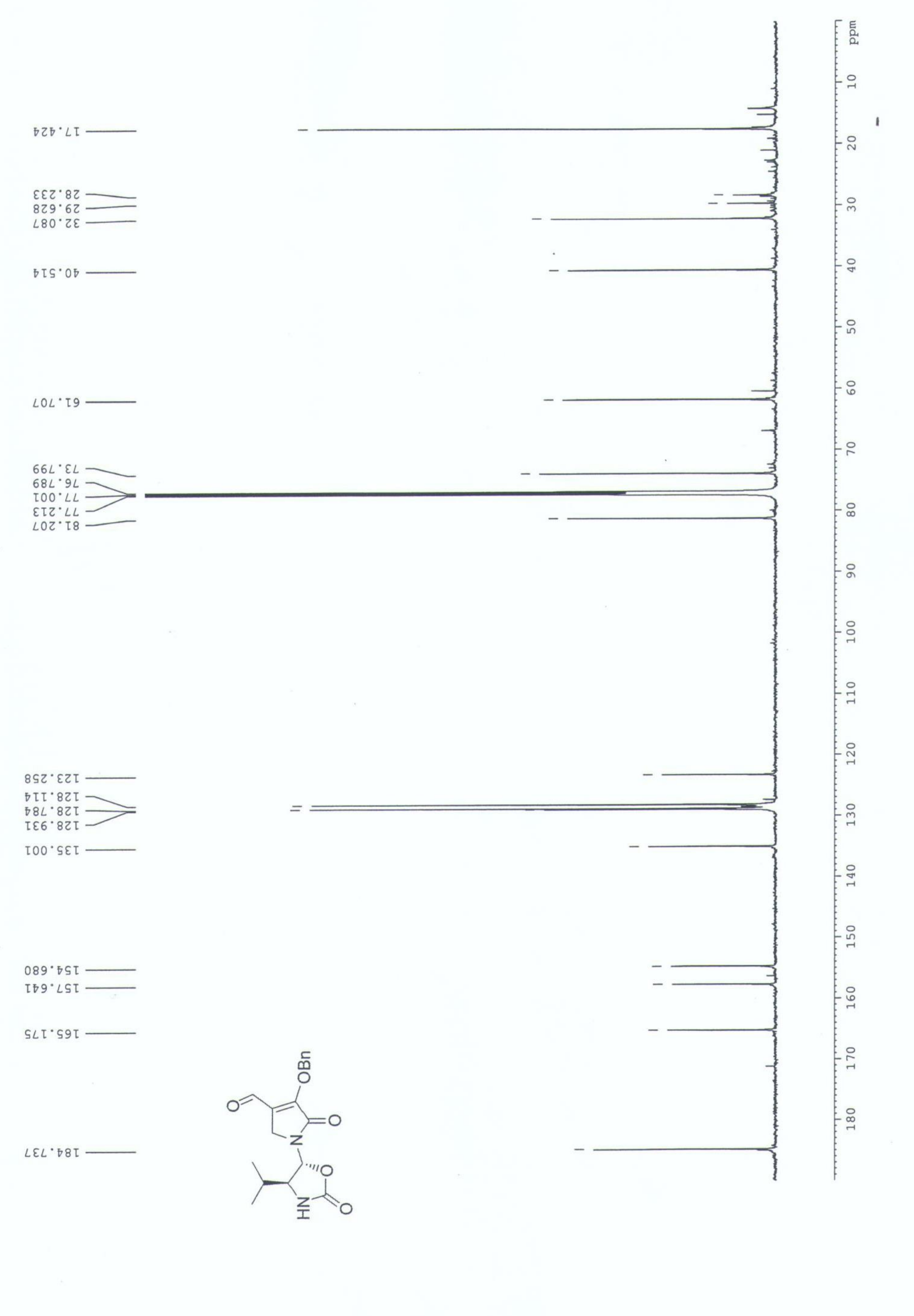

^{1}H NMR (600 MHz, $CDCl_3$) spectrum of compound **15**

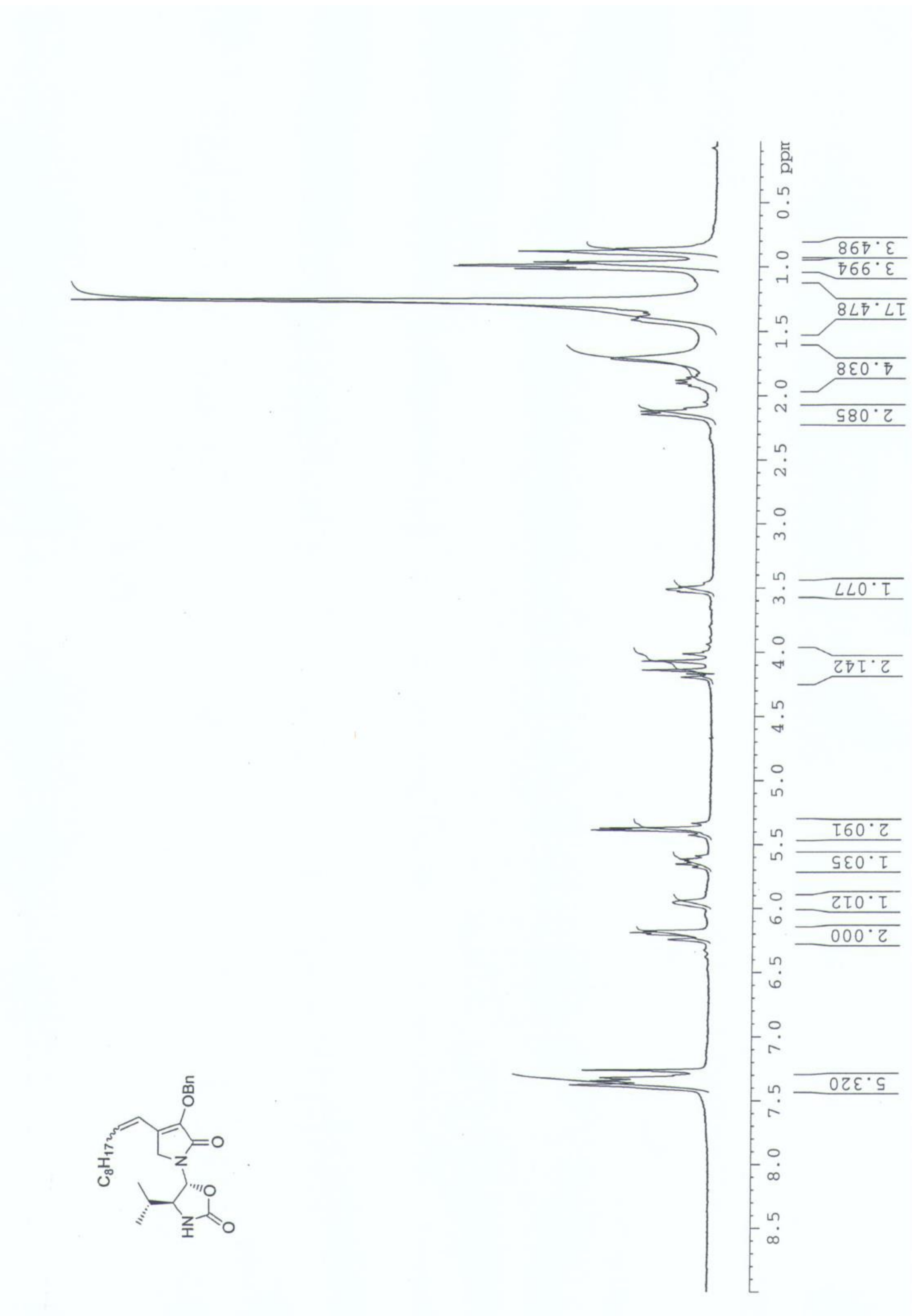

^{13}C NMR (600 MHz, $CDCl_3$) spectrum of compound **15** (mixture of stereoisomers)

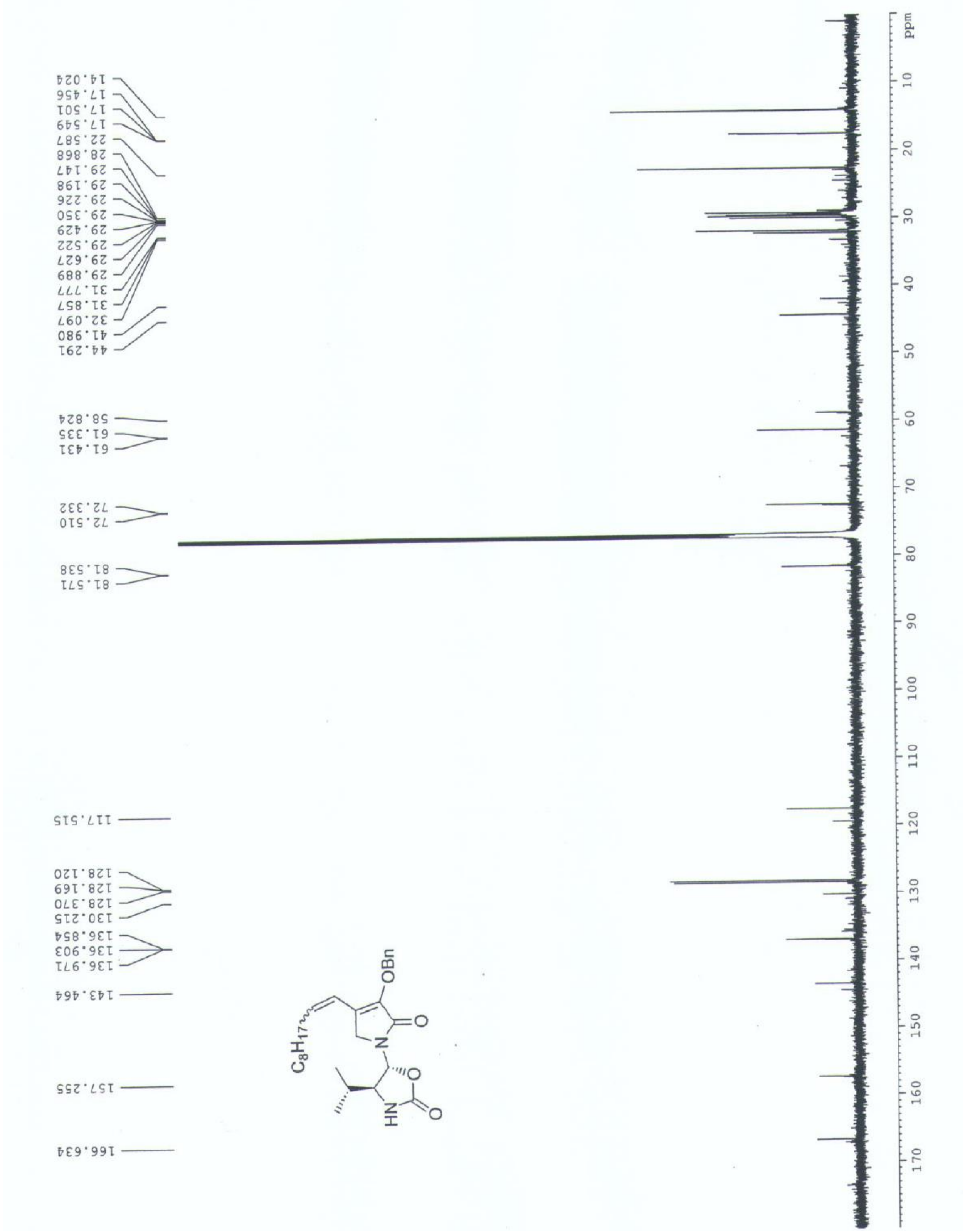

^{1}H NMR (300 MHz, CDCl3) spectrum of compound **16** (mixture of stereoisomers)

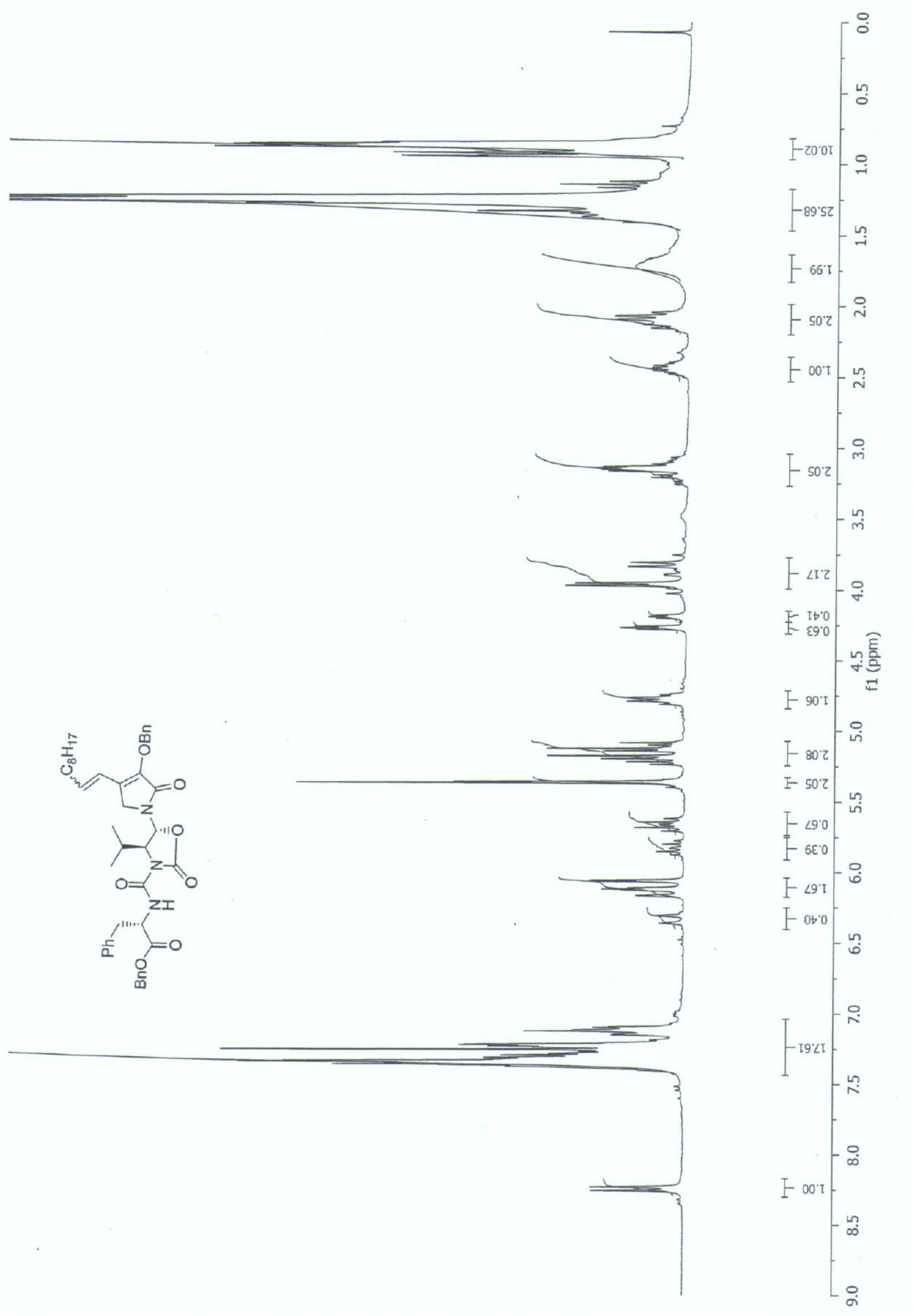

^{13}C NMR (300 MHz, $CDCl_3$) spectrum of compound **16** (mixture of stereoisomers)

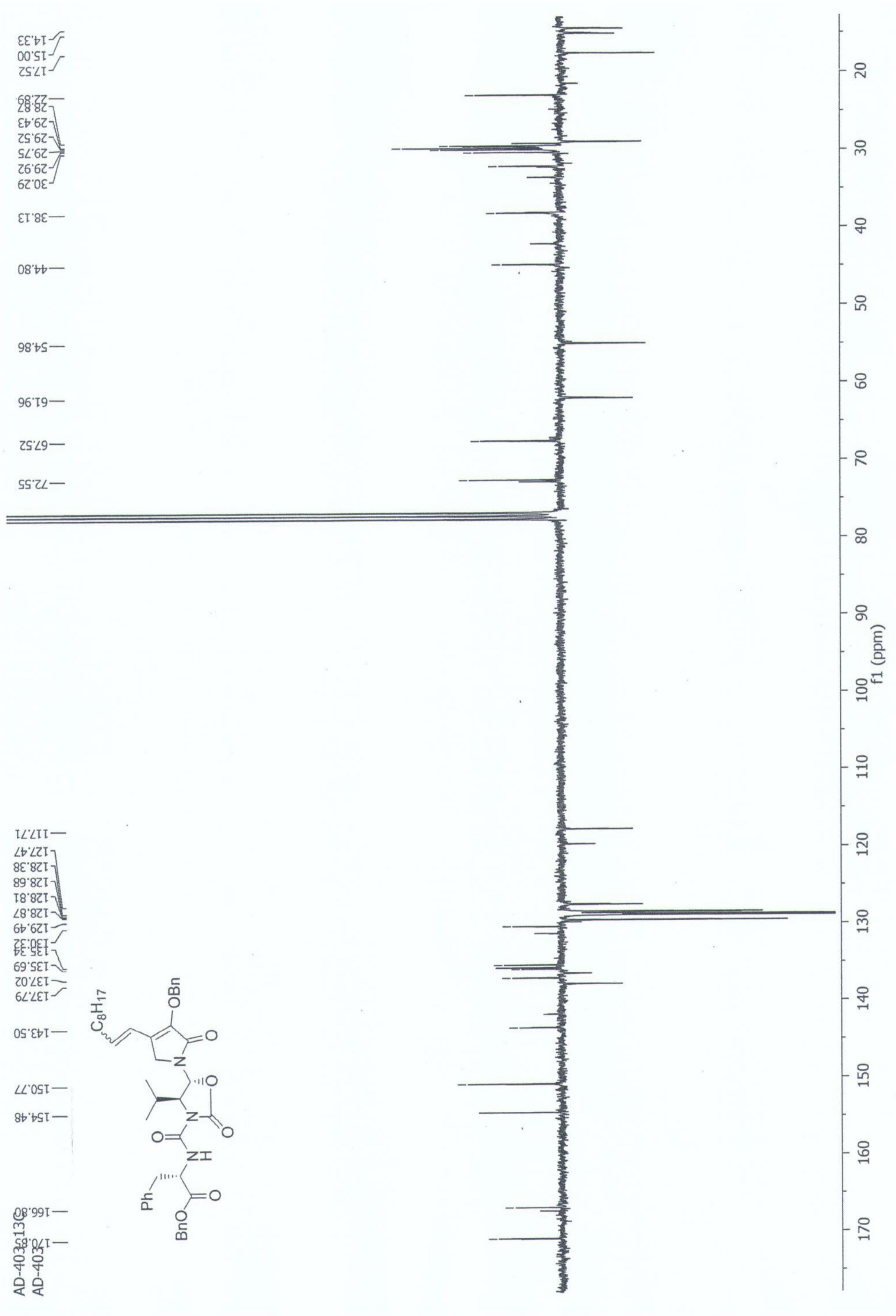

^{13}C NMR (300 MHz, $CDCl_3$) spectrum of compound **16** (mixture of stereoisomers, expanded)

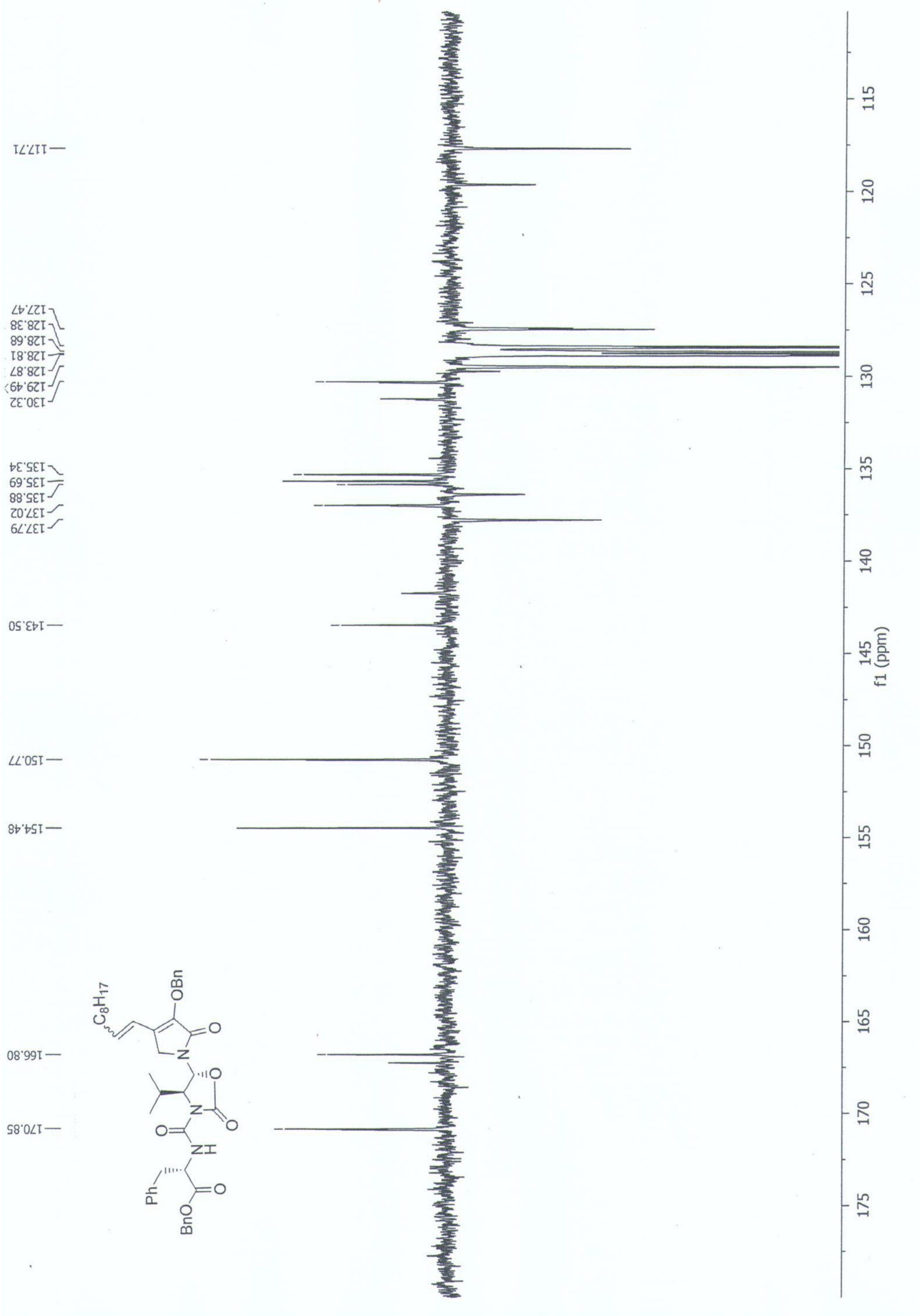

^{1}H NMR (600 MHz, DMSO-d_6) spectrum of compound **17**

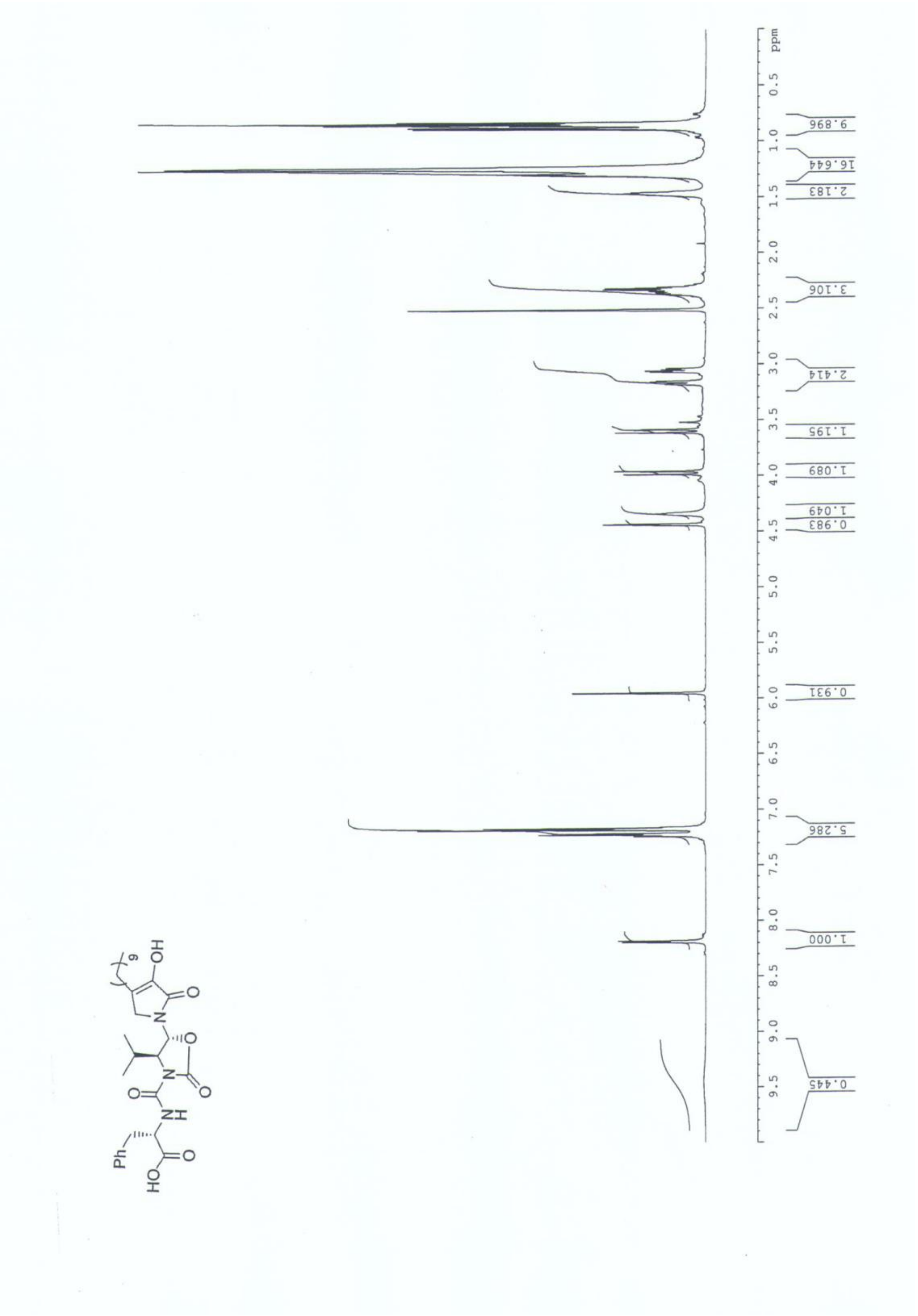

^{13}C NMR (600 MHz, DMSO-d_6) spectrum of compound **17**

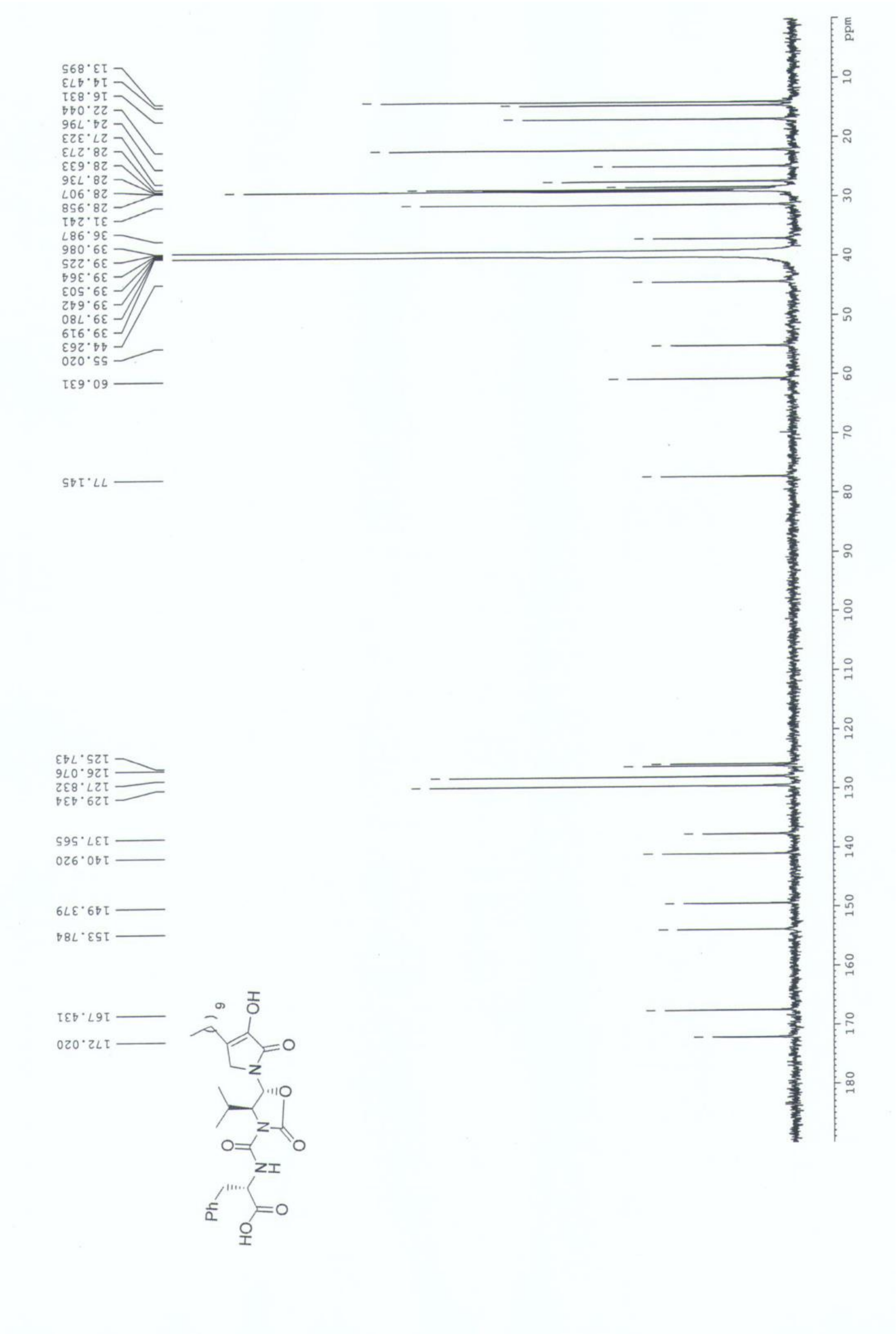

COSY (600 MHz, DMSO-d_6) spectrum of compound **17**

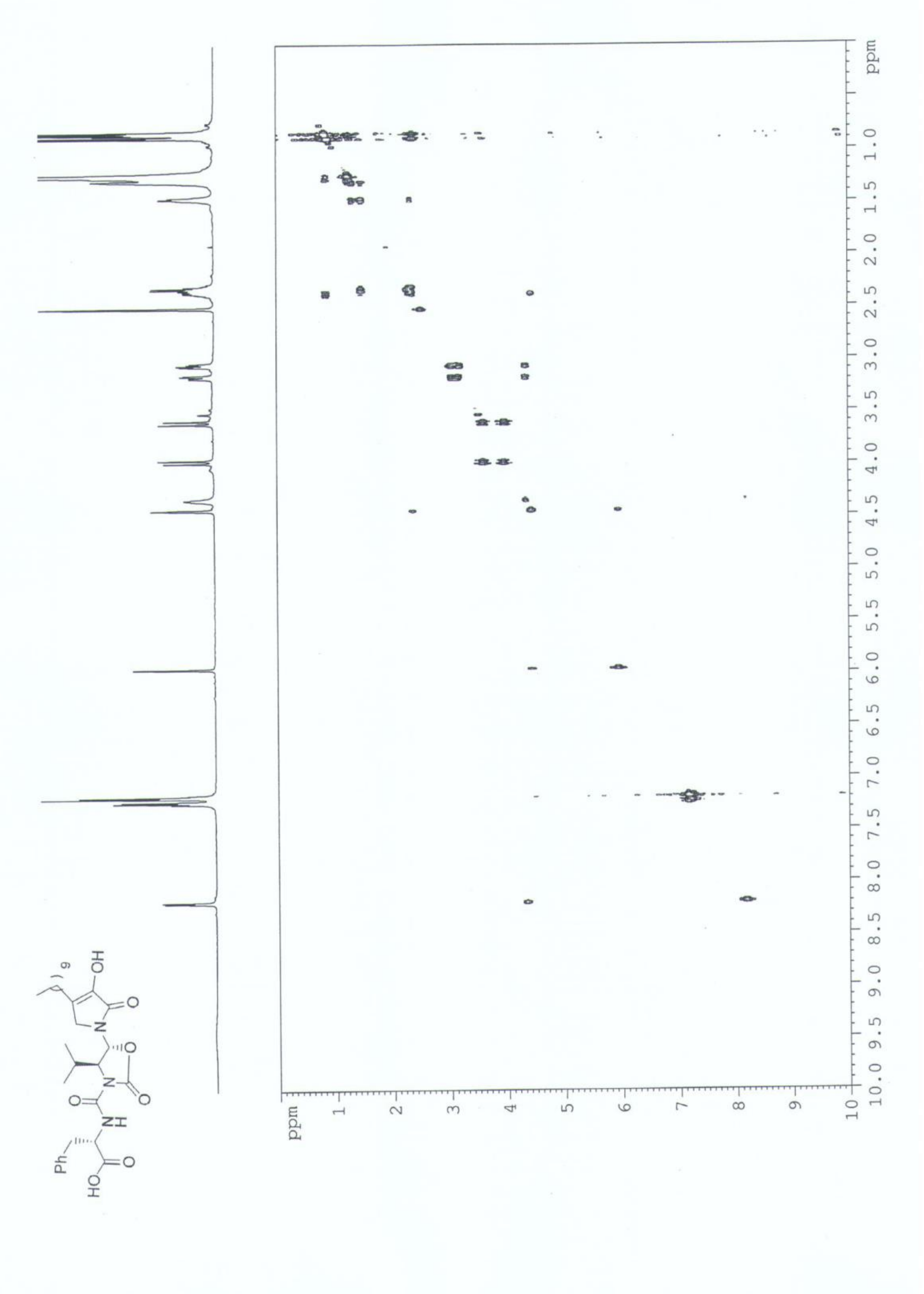

HSQC (600 MHz, DMSO-d_6) spectrum of compound **17**

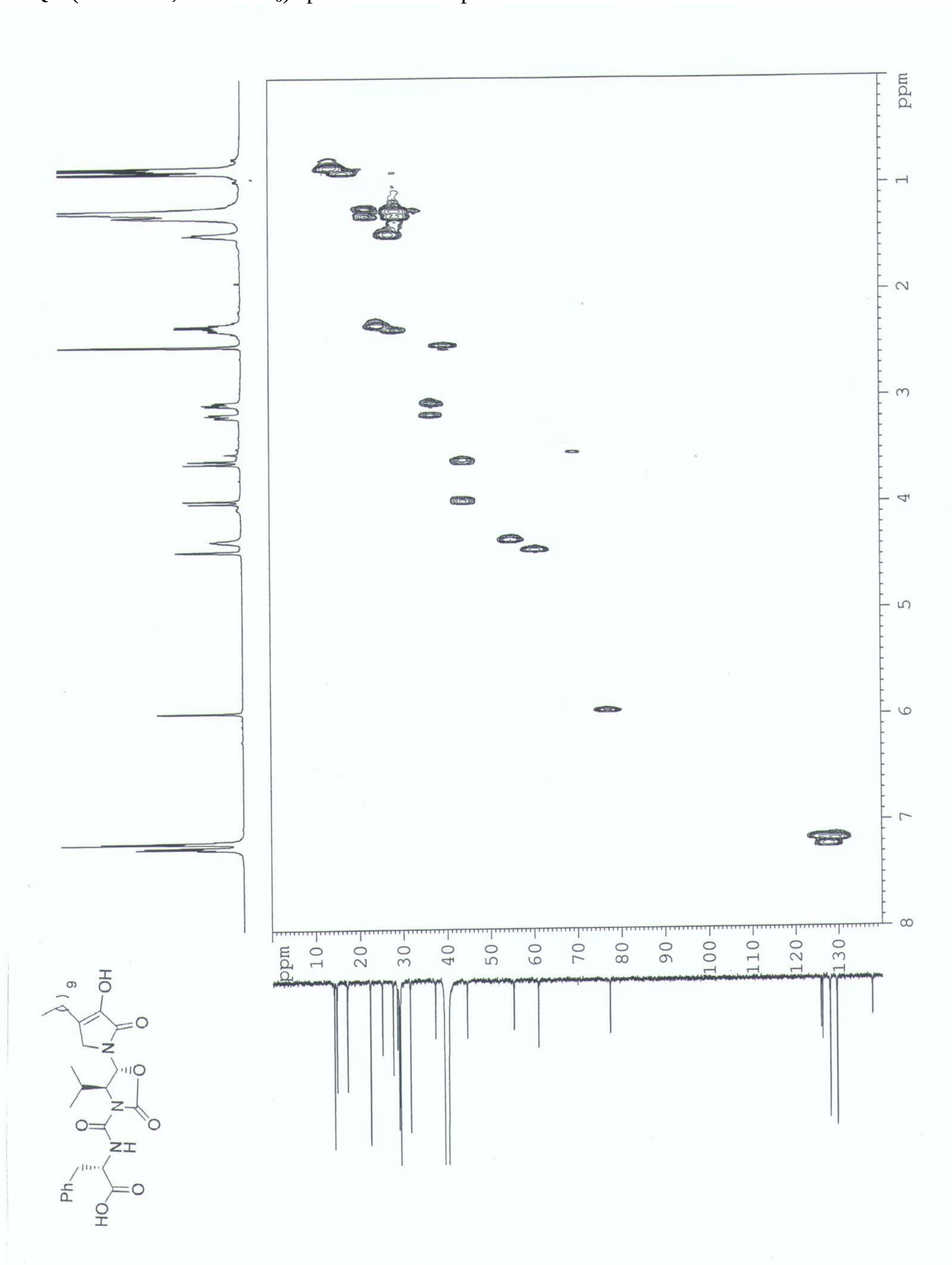

HMBC (600 MHz, DMSO-d_6) spectrum of compound **17**

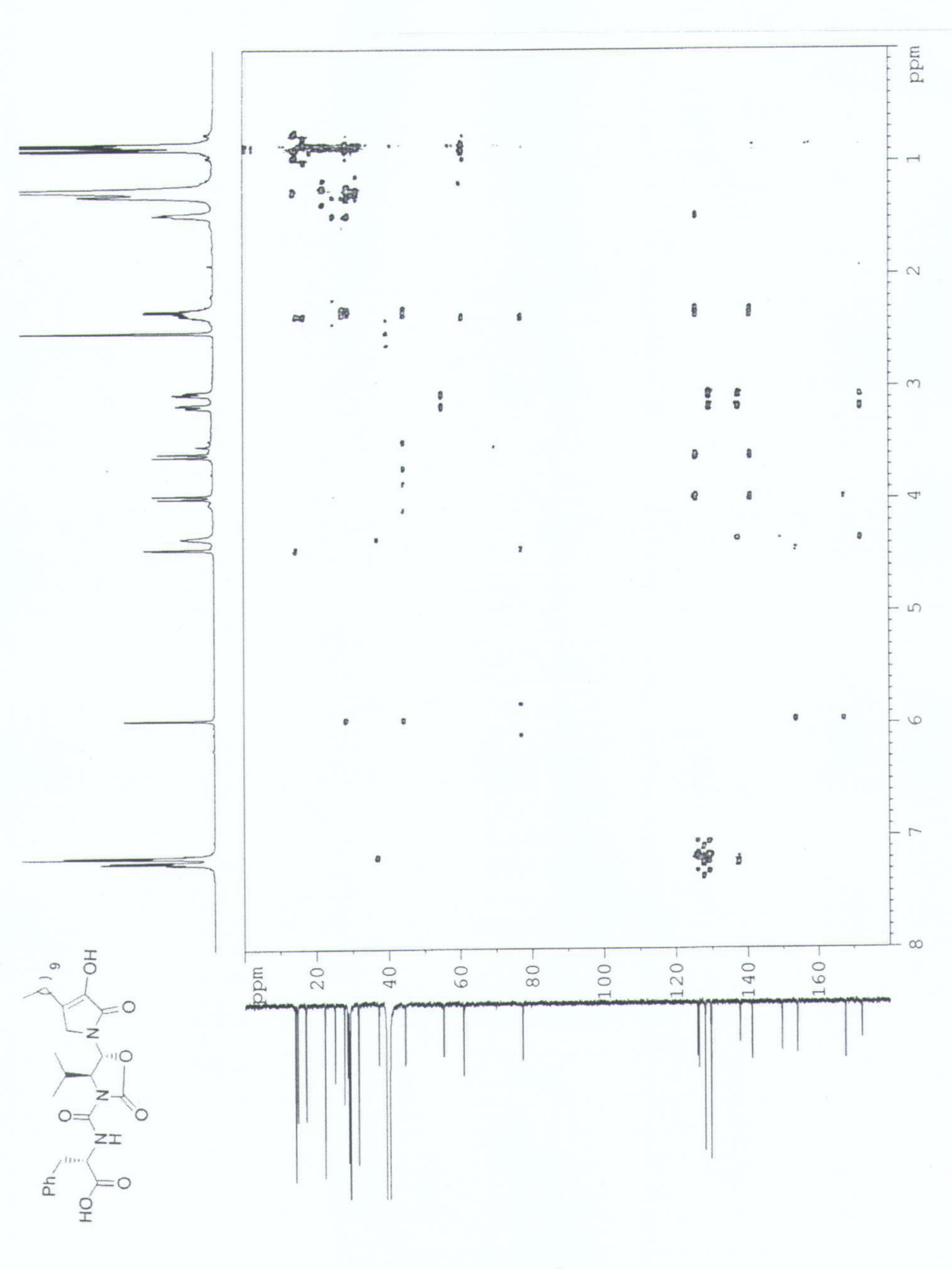

^{1}H NMR (600 MHz, DMSO-d_6) spectrum of compound **1**

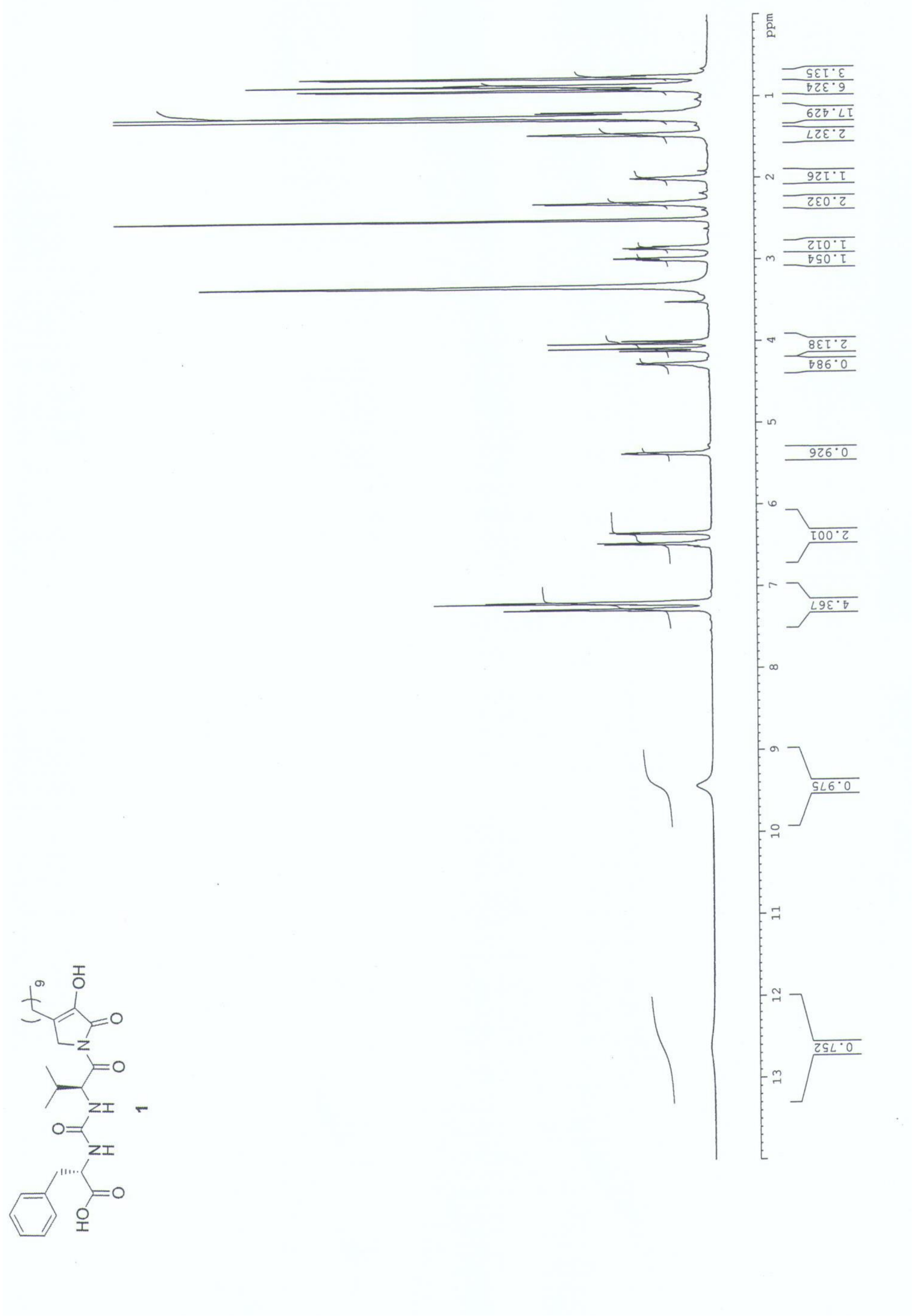

^{13}C NMR (600 MHz, DMSO-d_6) spectrum of compound **1**

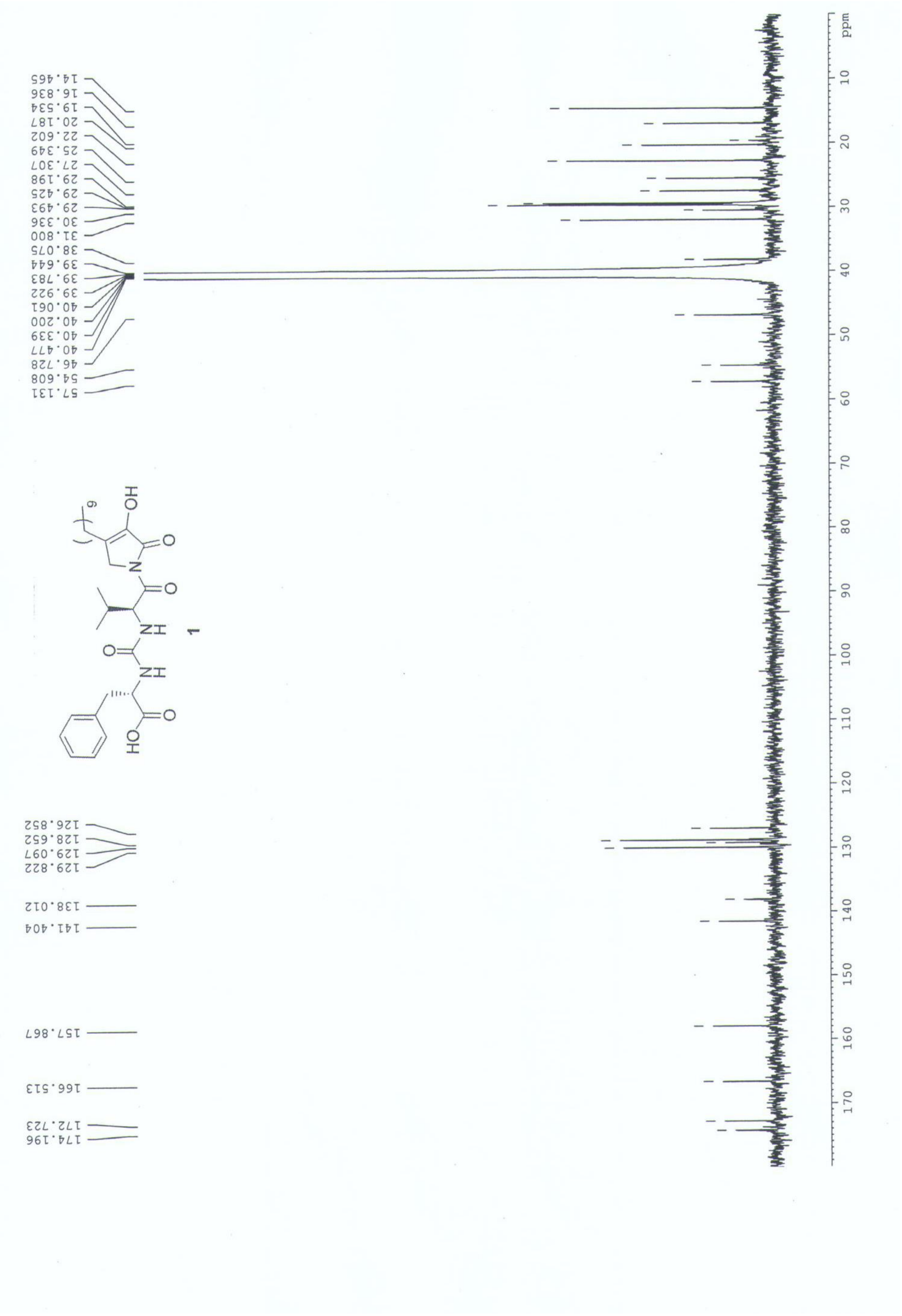

HSQC (600 MHz, DMSO-d_6) spectrum of compound **1**

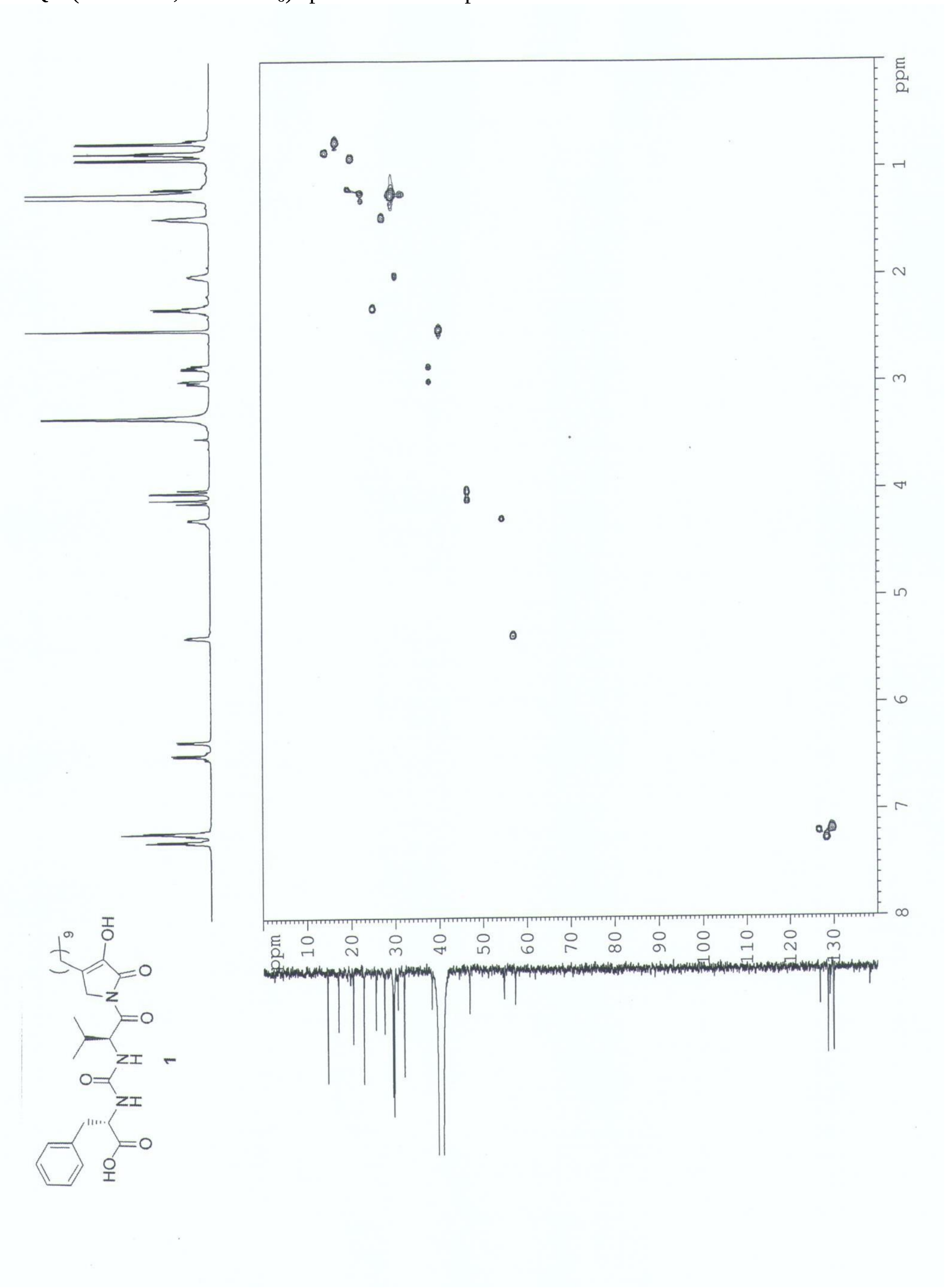

HMBC(600 MHz, DMSO-d_6) spectrum of compound **1**

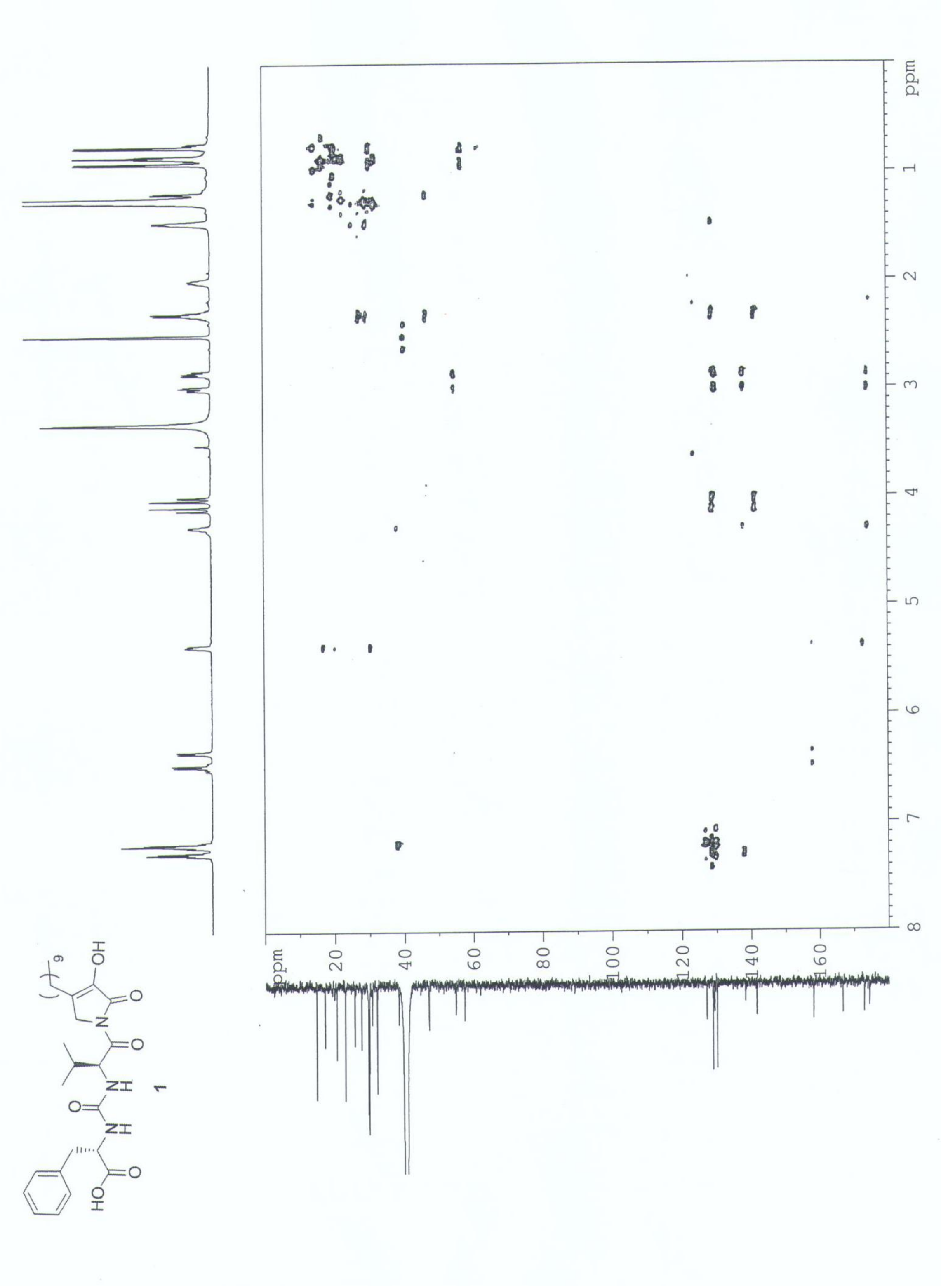

DEPT (600 MHz, DMSO-d_6) spectrum of compound **1**

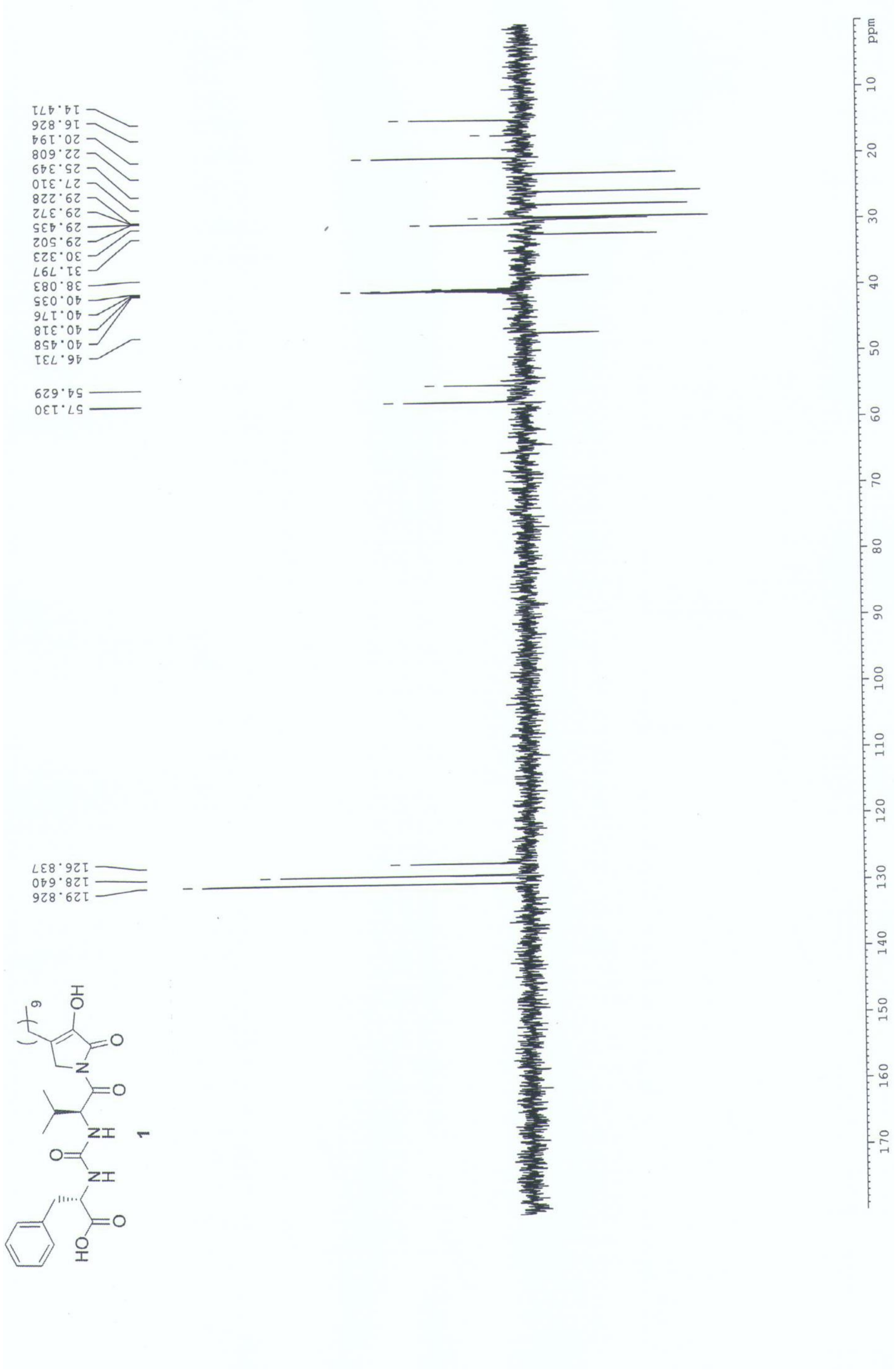

MIC of 1 against *Staphylococcus pseudintermedius* strains[a]

Strain	MIC (μg/mL)
1	16
2	16
3	16
4	8
5	16
6	16
7	16
8	16
9	16
10	16
11	16
12	16
13	16
14	16
15	16
16	16
17	16
18	16
19	16
20	16
21	16
22	16
23	16
24	16
25	16
ATCC 25923	16

[a]Sampled in Milano (Italy) in 2015

MIC of 17 against *Staphylococcus pseudintermedius* strains[a]

Strain	MIC (μg/mL)
1	16
2	32
3	64
4	64
5	32
6	32
7	32
8	64
9	64
10	64
11	32
12	32
13	32
14	32
15	32
16	32
17	64
18	64
19	64
20	32
21	32
22	32
23	32
24	32
25	64
ATCC 25923	32

[a]Sampled in Milano (Italy) in 2015

MIC of 1 against *Escherichia coli* strains

Strain	MIC (µg/mL)
1	128
2	128
3	128
4	128
5	128
6	128
7	128
8	128
9	128
10	128
11	128
12	128
13	128
14	128
15	128
16	128
17	128
18	128
19	128
20	128
ATCC 25923	128

MIC of 17 against *E. coli* strains

Strain	MIC (μg/mL)
1	128
2	128
3	128
4	128
5	128
6	128
7	128
8	128
9	128
10	128
11	128
12	128
13	128
14	128
15	128
16	128
17	128
18	128
19	128
20	128
ATCC 25923	128
